# Advances, Challenges, and Recommendations for Non-Destructive Testing Technologies for Wind Turbine Blade Damage: A Review of the Literature from the Past Decade

**DOI:** 10.3390/s26061773

**Published:** 2026-03-11

**Authors:** Guodong Qin, Yongchang Jin, Lizheng Qiao, Zhenyu Wu

**Affiliations:** 1 School of Mechanical Engineering, Zhejiang Sci-Tech University, Hangzhou 310018, China; 2 School of Intelligent Manufacturing, Jiaxing Vocational and Technical College, Jiaxing 314036, China; 3 Zhejiang Provincial Innovation Center of Advanced Textile Technology, Shaoxing 312030, China

**Keywords:** wind turbine blades, non-destructive testing, intelligent diagnostics, damage detection, predictive maintenance

## Abstract

As critical components of wind energy systems, the structural integrity of wind turbine blades is directly tied to the operational safety and economic performance of wind turbines. With blade designs trending toward larger and more flexible structures and operating environments becoming increasingly harsh, maintenance strategies must urgently shift from reactive approaches to predictive maintenance paradigms. From an engineering application perspective, this study conducts a systematic and critical review of non-destructive testing (NDT) and structural health monitoring (SHM) technologies for wind turbine blades. Drawing on the literature published over the past decade, we examine the field applicability, limitations, and engineering challenges of core NDT techniques—including vision-based methods, acoustic approaches, vibration analysis, ultrasound, and infrared thermography. Particular emphasis is placed on the integration of data-driven approaches with engineering practice, evaluating the role of machine learning in fault classification and anomaly diagnosis, as well as the contributions of deep learning to automated defect detection in image and signal data. Moreover, this paper critically discusses the growing use of robotic inspection platforms, such as unmanned aerial vehicles and climbing robots, as multi-sensor carriers enabling rapid and comprehensive blade assessment. By comparatively analyzing detection performance, cost, and automation levels across technologies, we identify key engineering barriers, including environmental noise robustness, signal attenuation within complex blade structures, and the persistent gap between laboratory methods and field deployment. Finally, we outline forward-looking research directions, encompassing multi-modal sensor fusion, edge computing for real-time diagnostics, and the development of standardized SHM systems aimed at supporting full lifecycle blade management.

## 1. Introduction

### 1.1. Research Background and Significance

Driven by global energy transition efforts and the pursuit of carbon peak and carbon neutrality targets, wind energy—one of the cleanest and most renewable energy sources—has been experiencing rapid growth in its development and utilization [1]. As the core equipment responsible for wind-to-electricity conversion, the reliability and stability of wind turbines directly influence the economic performance of wind farms [2]. Among the various components of a wind turbine, the blade serves as the key aerodynamic element for capturing wind energy. Its length has continuously increased to enhance power generation efficiency [3], making it not only one of the most expensive components but also one of the most vulnerable. The manufacturing cost of the blades alone can account for up to 22.2% of the entire turbine system [4].

**Figure 1:**
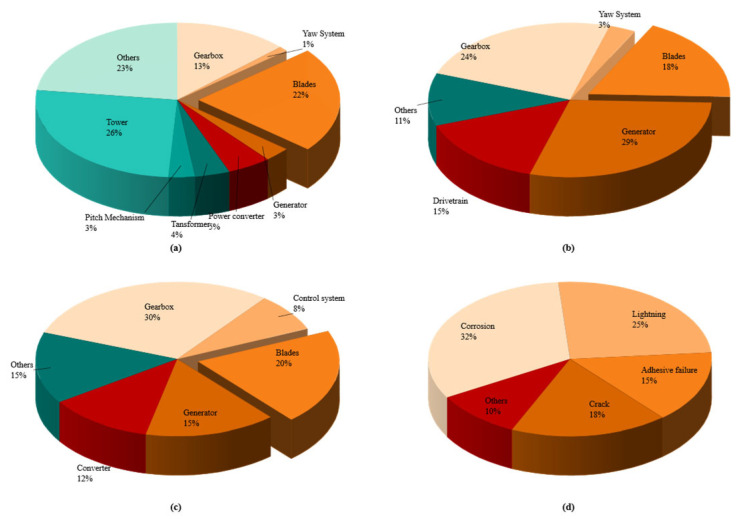
Costs and repair expenses. (**a**) The manufacturing cost ratio of the entire wind power system. Based on data retrieved from Ref. [4]. (**b**) The proportion of downtime for each component of the entire wind power system. Based on data retrieved from Ref. [5]. (**c**) The maintenance cost of each component of the entire wind power system. Based on data retrieved from Ref. [6]. (**d**) The proportions of types of damage to wind turbine blades. Based on data retrieved from Ref. [6].

Throughout their 20–25-year design life, blades are subjected to complex cyclic loads—including aerodynamic, gravitational, and inertial loads—as well as harsh environmental conditions such as strong winds, lightning strikes, rain erosion, ultraviolet radiation, and marine salt-spray corrosion [5]. As illustrated in Figure 1, blade-related downtime accounts for 18% of the total turbine downtime and blade maintenance accounts for 20% of the total maintenance cost, both ranking first among all turbine components [6]. These long-term service conditions make composite blades particularly prone to fatigue-induced cumulative damage, leading to their being one of the components with the highest failure rates in wind turbines [6].

Once severe blade failures occur, they not only result in costly repairs or replacements and substantial revenue losses due to downtime but may also lead to catastrophic events such as blade fracture or tower collapse, posing significant threats to the safety of the entire wind farm [7]. Therefore, developing efficient and accurate blade condition monitoring and non-destructive testing technologies—capable of achieving early detection and diagnosis of damage—is of vital theoretical and engineering importance for ensuring reliable turbine operation, reducing operation and maintenance costs, extending equipment lifespan, and enhancing the competitiveness of wind energy [8].

### 1.2. Common Types of Damage to Wind Turbine Blades and Their Causes

Wind turbine blades (WTBs) are predominantly manufactured from glass-fiber-reinforced polymer (GFRP) or carbon-fiber-reinforced polymer (CFRP) composites, whose anisotropic, heterogeneous nature and harsh service environment give rise to complex and diverse damage mechanisms [9]. Table 1 summarizes the major categories of blade damage according to their locations and failure modes.

**Figure 2:**
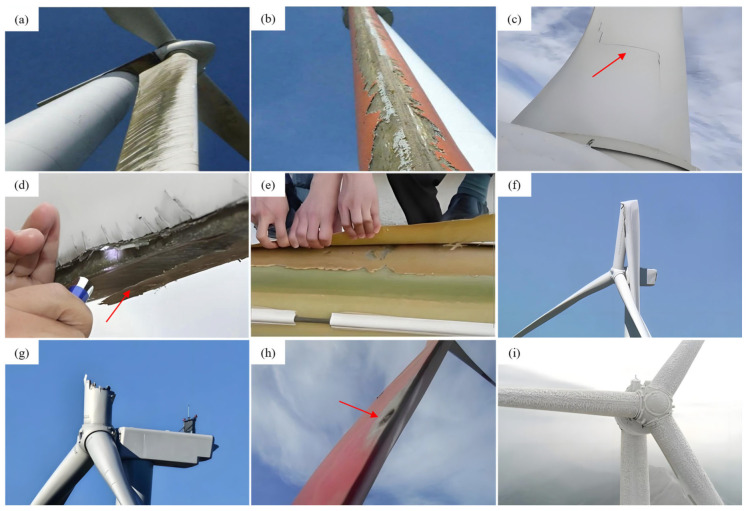
WTB surface damage diagrams. (**a**) Leading-edge erosion. (**b**) Coating detachment. (**c**) Crack. (**d**) Debonding. (**e**) Delamination. (**f**) Spar-cap fracture. (**g**) Shear-web buckling. (**h**) Lightning strikes. (**i**) Ice accumulation. (Images compiled by the authors from open online sources for illustrative purposes).

Surface damage primarily includes leading-edge erosion and surface cracking. Leading-edge erosion (Figure 2a)—one of the most frequently observed forms of degradation—is typically induced by high-velocity impacts from raindrops [10], hail [11], and sand particles [12], which progressively remove protective coatings and deteriorate the substrate, as shown in Figure 2b. This form of erosion can markedly alter the aerodynamic profile of the blade and reduce overall power generation efficiency [5]. Surface cracks (Figure 2c), by contrast, generally originate from manufacturing defects or external impacts [13].

Internal damage, such as debonding (Figure 2d) and delamination (Figure 2e), represents another critical category of defects. These failures often arise from improper manufacturing processes or in-service loadings—including impact and fatigue—that weaken interlaminar bonding strength [14]. Such defects are highly concealed in the early stages but can result in rapid deterioration of structural stiffness and load-carrying capacity once initiated [17].

Severe structural failures, including shear-web buckling (Figure 2f) and spar-cap fractures (Figure 2g), constitute some of the most consequential forms of damage. These failures are generally triggered by extreme loading events or by the accumulation of fatigue damage over long-term operation, and they can lead to abrupt loss of structural integrity, posing major risks to overall turbine safety [15]. The impact of these factors can be further amplified by regional climatic conditions; for instance, studies have shown that blade failure mechanisms are closely linked to specific environments, such as typhoon-prone zones [18] or regions with distinct seasonal weather patterns [19].

Environmental factors further contribute to blade degradation. The most common environmental hazards are lightning strikes (Figure 2h) and ice accretion (Figure 2i). Lightning—an especially significant threat in both offshore and onshore wind farms—can cause perforation, ablation, or even internal explosions due to the instantaneous high temperature and pressure generated during discharge [20]. Ice accretion, on the other hand, increases blade mass, disrupts aerodynamic balance, and may induce severe vibrations during turbine operation [11].

These damage modes rarely occur in isolation; instead, they often interact and co-evolve. Surface cracks, for instance, may propagate inward and develop into delamination, whereas stress concentration around delaminated regions can accelerate the initiation of new cracks [13]. Consequently, the ability to effectively detect and monitor multiple, simultaneously evolving damage types remains a central challenge in blade health management.

### 1.3. Importance of NDT in Wind Turbine Blade Health Monitoring

**Figure 3:**
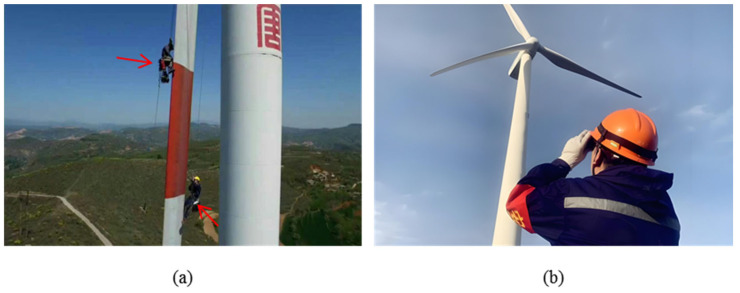
Manual testing method for wind turbine blades. (**a**) Manual inspection with access equipment. (**b**) Ground-based visual inspection.

Traditional manual inspection methods used in blade health monitoring suffer from significant limitations—including high labor intensity, low inspection accuracy, and the inability to achieve comprehensive coverage [20]—as illustrated in Figure 3. As a result, non-destructive testing technologies, which enable real-time assessment of material and structural conditions without causing damage, have become essential tools for monitoring the health state of wind turbine blades. Compared to manual inspection, NDT technologies offer several distinct advantages:

1. Real-time monitoring

NDT techniques can continuously acquire data during turbine operation, allowing early detection of damage or potential failures [21]. In particular, non-contact methods such as acoustic emission (AE) and infrared thermography enable dynamic monitoring without interrupting normal turbine operation [20].

2. High sensitivity and accuracy

NDT methods—including ultrasonic testing [22] and AE analysis [23]—exhibit high sensitivity to early-stage defects, enabling the detection of micro-cracks and minor damage. This capability provides timely warnings and helps prevent catastrophic structural failures [24].

3. Broad applicability

Different NDT approaches, such as ultrasound [22], AE [23], and vibration analysis [25], are suitable for identifying a wide range of damage types. By integrating multiple NDT techniques, a comprehensive assessment of blade health can be achieved, covering both surface and internal defects, as well as structural and operational abnormalities.

4. Reduced cost and improved efficiency

By minimizing the need for labor-intensive manual inspections and reducing turbine downtime, NDT technologies greatly enhance monitoring efficiency and accuracy. This, in turn, lowers maintenance costs, reduces operational interruptions, and improves the overall economic performance and reliability of wind turbine systems [7].

### 1.4. Review Scope, Methodological Framework, and Main Highlights

**Figure 4:**
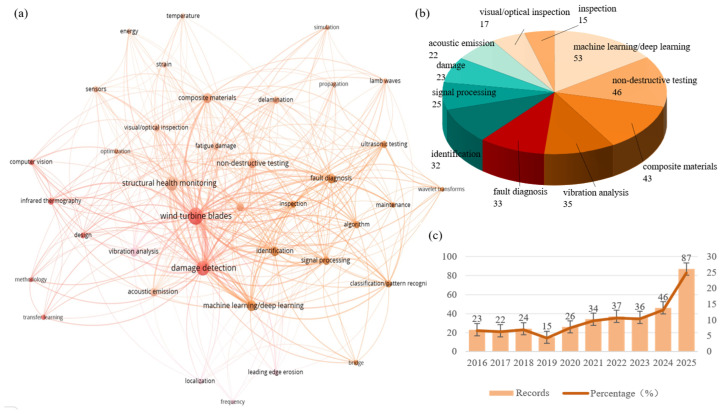
Bibliometric analysis of wind turbine blade NDT research (2016–2025). (**a**) Distribution of research hotspots based on keywords. (**b**) Distribution of research hotspots in wind turbine blade NDT based on keyword occurrences. (**c**) Annual number of publications and their corresponding percentage of the total.

To provide a comprehensive overview of the research landscape, a bibliometric analysis was conducted based on publications indexed in the Web of Science core collection from 2016 to 2025. Figure 4 presents the key findings of this analysis. Specifically, Figure 4a illustrates the distribution of keywords related to material properties, physical quantities, and sensing elements, highlighting the predominant focus on parameters such as temperature, energy, and strain. Figure 4b focuses on the distribution of specific NDT techniques and data analysis methods, where machine learning/deep learning emerges as the most prominent category, followed by traditional techniques like acoustic emission and ultrasonic testing. Figure 4c shows the annual number of publications and their corresponding percentage of the total over the same period, revealing a steady growth in research output and underscoring the increasing attention devoted to this field. These visualizations collectively depict the evolution and current hotspots of wind turbine blade NDT research, setting the stage for the detailed technology review that follows.

**Figure 5:**
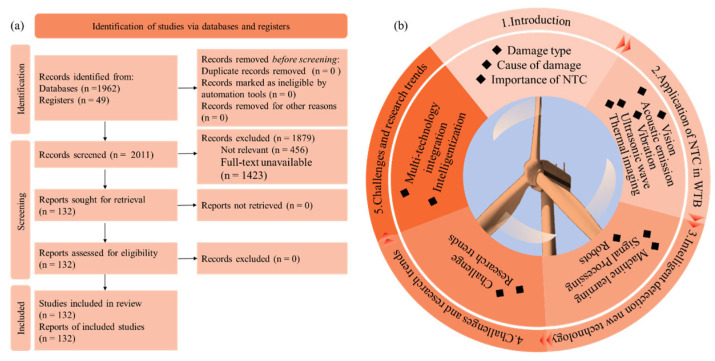
Research framework and literature sources. (**a**) PRISMA 2020 flow diagram of the study selection process. (**b**) Organizational structure of the paper.

This article aims to provide a systematic review and critical assessment of non-destructive testing and SHM technologies for wind turbine blades, with particular emphasis on recent advances integrating traditional approaches with emerging intelligent techniques. This review was conducted in accordance with the Preferred Reporting Items for Systematic Reviews and Meta-Analyses (PRISMA) 2020 guidelines (Supplementary Materials). A systematic literature search was performed in the Web of Science Core Collection using a comprehensive search strategy covering traditional and emerging NDT techniques, supplemented by additional searches in other databases and manual screening of reference lists. A total of 132 studies were included in the final review. The PRISMA flow diagram illustrating the study selection process is provided in Figure 5a. The overall structure of this paper is illustrated in Figure 5b.

To address the gaps in the existing literature, this review offers the following distinct contributions:

1. Scenario-based comparative framework. Unlike prior reviews that focus on individual techniques, we systematically compare NDT methods across onshore/offshore environments and manufacturing/in-service stages, providing practical guidance for technology selection based on environmental robustness, cost, and deployment feasibility.
2. Integration of traditional and intelligent paradigms. While existing surveys often treat conventional and AI-driven methods separately, we explicitly analyze their convergence.
3. Engineering implementation and hybrid strategies. Moving beyond laboratory discussions, we synthesize real-world engineering cases that demonstrate quantifiable benefits.
4. Actionable research roadmap with standardization emphasis. Instead of generic future directions, we identify specific priorities—multi-modal fusion, few-shot learning, and SHM standardization—highlighting certification as a critical bridge between research and industrial adoption.

## 2. Application of Traditional NDT Technologies in Wind Turbine Blades

Although emerging technologies continue to appear, traditional NDT methods still play an irreplaceable role in the condition assessment of wind turbine blades due to their relative maturity and clear principles. These methods cover multiple inspection dimensions, from macroscopic morphology to microscopic structure and from surface to internal defects.

### 2.1. Visual and Optical Inspection Methods

Visual and optical inspection methods are the most intuitive means for detecting defects in wind turbine blades, primarily assessing the surface condition and geometric morphology of blades through images or optical signals [16], thereby inspecting blade damage or defects.

#### 2.1.1. Aerial Photography and Digital Image Correlation Technology

Traditional methods for inspecting wind turbine blade defects, such as manual climbing or basket inspections, suffer from low efficiency and high risks [26]. However, the introduction of unmanned aerial vehicle (UAV) aerial photography technology has revolutionized this landscape [27]. Equipped with high-definition cameras, UAVs can capture high-resolution images of blade surfaces at close range and from multiple angles [28] and are particularly suitable for detecting surface defects such as cracks, leading-edge erosion, and lightning damage [29]. Based on these images, combined with deep learning algorithms, automatic defect identification and classification can be achieved [30], significantly enhancing the automation level and accuracy of inspection [31].

A recent study employed a Digital Image Correlation (DIC) system integrated on an unmanned aerial vehicle to capture the dynamic response of operating wind turbine blades [32]. Using a stereo imaging setup combined with dynamic image-stitching algorithms, the approach enables high-fidelity measurement of full-blade deformation and vibration characteristics during rotation. Experimental results show that the method delivers sufficient robustness and accuracy for remote, real-time structural condition monitoring in both offshore and onshore wind farms, highlighting its promise as a field-deployable SHM solution.

**Figure 6:**
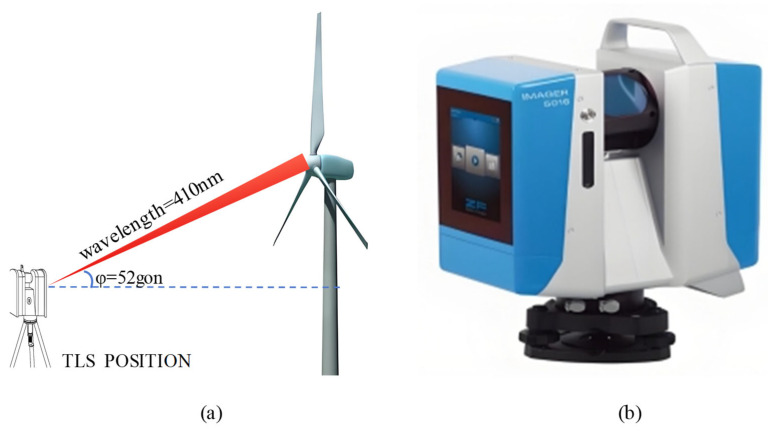
Detection method and equipment described by Stałowska et al. [33]. (**a**) Detection method. (**b**) Detection equipment.

DIC technology is more suitable for precise blade measurement scenarios. As shown in Figure 6, to evaluate the capability of Terrestrial Laser Scanning (TLS) in detecting blade surface defects, Stałowska et al. [33] found that at an optimal incident angle of 52° and a specific wavelength of 410 nm, its detection effectiveness is comparable to photogrammetry, providing a powerful tool for the quantitative assessment of blade geometric accuracy and surface condition, although it has limitations in detecting blade edge defects. It should be noted that the TLS used in this study is of the Phase-Shifting type, rather than a time-of-flight system.

A visual inspection strategy based on image-processing techniques—referred to as the three-point slope deviation method—has also been developed to assess the operational condition of wind turbine blades [34]. The approach employs a sequence of image calibration, stitching, preprocessing, and threshold-segmentation procedures to extract geometric features from high-quality blade images. Experimental evaluations indicate that the method achieves high defect recognition accuracy and demonstrates robust monitoring performance, suggesting its suitability for practical blade status assessment in field applications.

**Figure 7:**
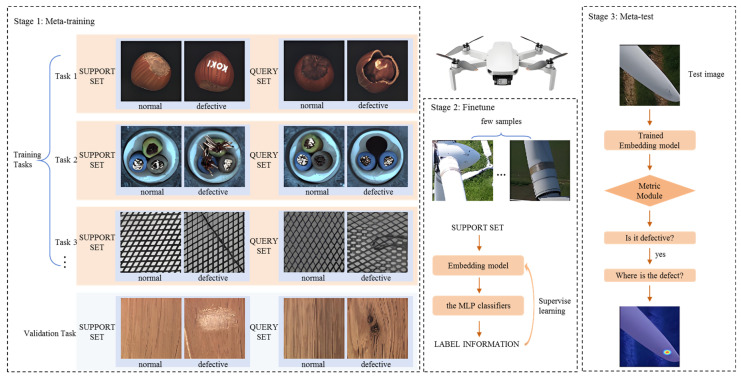
The third-order processing process described by Yu et al. [35].

Addressing the challenges of insufficient samples, poor detection accuracy for small targets, and concealed defects in wind turbine blade surface defect detection, Yu et al. [35] proposed an innovative meta-learning-inspired method, as shown in Figure 7. Their innovation introduces a real-time feedback mechanism in the model training loop through third-order processing, combined with dynamic activation mapping to enhance the detection capability for small-scale defects. Experimental results indicate that this method significantly improves the recognition capability for blade surface defects under few-shot training conditions.

To solve the problem of achieving fast and accurate damage detection through image data, Guo et al. [36] proposed a blade damage identification framework based on Deep Convolutional Neural Networks (CNNs), which performed excellently in identifying multiple damage types, validating the application prospects of deep learning in structural health monitoring.

#### 2.1.2. Three-Dimensional Photogrammetry and Laser Scanning Technology

For large wind turbine blades, the conformity of their overall geometric shape to the design model is crucial. Three-dimensional photogrammetry technology uses a series of two-dimensional images taken from different angles to reconstruct a three-dimensional model of the object through algorithms. Ozbek et al. [37] used four CCD cameras on a 2.5 MW wind turbine to successfully measure the dynamic deformation of the rotor from 220 m away, with an average accuracy of ±25 mm, and identified some important modes, demonstrating the feasibility of this technology in monitoring large structures.

**Figure 8:**
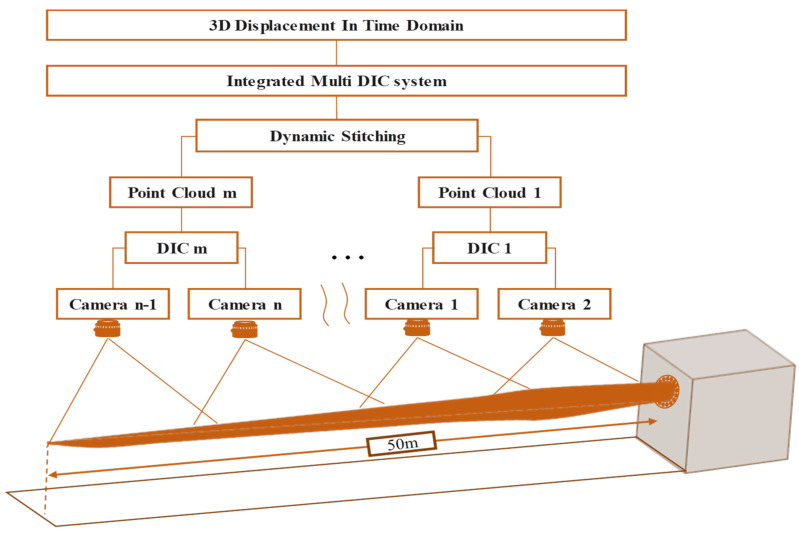
Multiple independent standard stereo vision systems using multi-camera DIC [38].

Multi-camera measurement systems with dynamic spatial data stitching technology have been applied in wind turbine blade inspection. Poozesh et al. [38] used two stereo vision systems on a 50 m long utility-scale blade for quasi-static and cyclic loading experiments, as shown in Figure 8, achieving distributed displacement and strain measurements over the entire utility-scale blade surface area, breaking through the limitation of traditional methods that can only measure at limited points. Wu et al. [39] used DIC technology, and further research showed that optical technology can effectively monitor the full-field deformation of operating blades and successfully identify local strain concentrations caused by cracks, providing rich data for the mechanical state assessment of blades.

#### 2.1.3. Summary of Visual and Optical Inspection Methods

In this section, the advantages and disadvantages identified by various researchers in the application of visual and optical inspection methods in wind turbine blades are shown in Table 2.

In summary, visual and optical inspection technologies, accompanied by the integration of UAV technology and 3D photogrammetry, have broad application prospects in the surface defect detection and monitoring of wind turbine blades. However, it is important to acknowledge the inherent bottlenecks these methods face in practical applications. Their performance is heavily dependent on lighting conditions and weather, with fog, rain, or low-light environments significantly degrading image quality. Furthermore, these techniques are fundamentally limited to surface defect detection and are unable to provide information about internal damage such as delamination or debonding. Addressing these limitations will be crucial for future development. In the future, combined with multi-modal measurement and advanced algorithms, these technologies are expected to achieve more precise detection capabilities, providing fundamental data support for wind turbine blade defect detection.

### 2.2. Acoustic Testing Methods

Acoustic technology, as a means of real-time monitoring of material damage, has achieved remarkable results in the health monitoring of wind turbine blades. As the scale and power rating of wind turbine units continue to increase, problems such as fatigue damage and crack propagation in blades are becoming increasingly severe, making AE technology play an important role in detecting early damage and real-time monitoring [17].

#### 2.2.1. AE Technology for Crack and Delamination Monitoring

**Figure 9:**
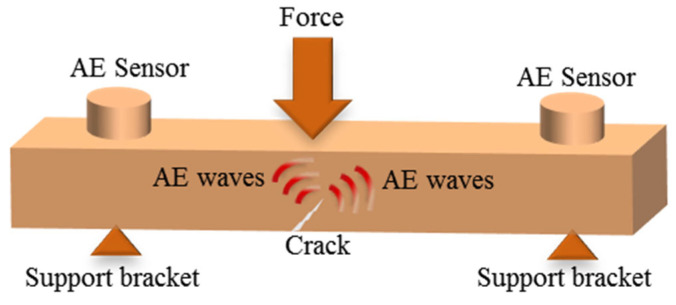
Schematic diagram of AE detection.

AE technology is a dynamic NDT method. The principle is shown in Figure 9. When external forces act on the material being inspected, micro-cracks, inclusions, voids, and other defects inside undergo deformation or expansion, generating transient elastic waves. These signals are collected using piezoelectric sensors. Subsequently, AE events are classified and located based on characteristic parameters, and the damage degree and evolution trend of the material are analyzed [17].

Researchers have conducted extensive research on AE detection in wind turbine blades. For example, in terms of the theoretical foundation for acoustic monitoring of composite blades, Pang et al. [40] studied the attenuation characteristics and source locations of AE signals in resin-based composites. Zhao et al. [41] conducted the first research on material damage monitoring based on AE in China. By comparing AE signals with vibration signals in different damage states, they verified the superiority of AE in damage identification, showing its potential in early fault detection. Currently, this technology is increasingly being adopted in the field for wind turbine blade operation and maintenance monitoring [42].

In terms of monitoring the initiation and propagation of delamination and cracks using AE sensor arrays, Tang et al. [23,43] conducted fatigue tests on a full-scale blade 45.7 m long. Using a pattern recognition method based on K-means clustering, they finely classified AE activities corresponding to different damage mechanisms. Experimental results showed that the intensity of AE signals significantly increased at the blade root and high-load areas, indicating a higher frequency of damage occurrence.

**Figure 10:**
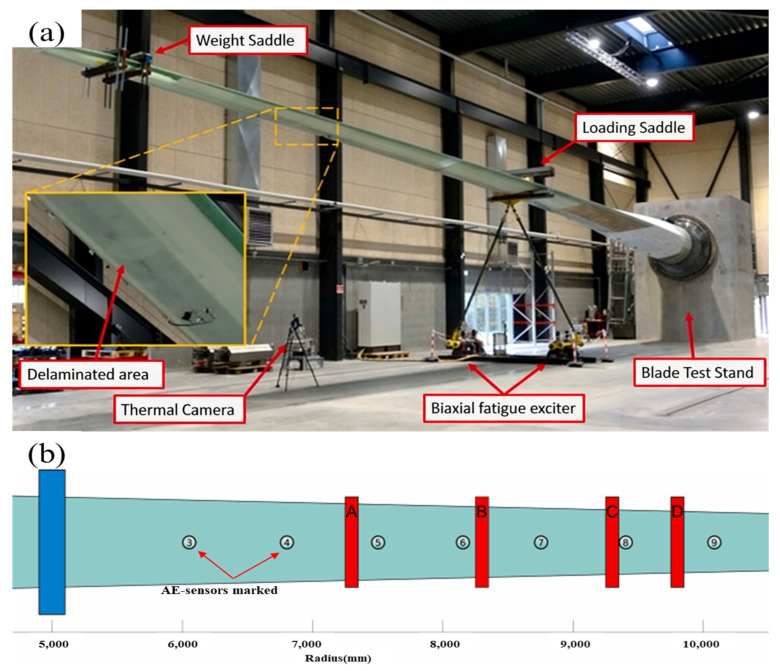
Detection device and data described by Mielke et al. [44]. (**a**) Full-scale fatigue load testing device for wind turbine blades. (**b**) Schematic of the 14.3 m blade with the AE sensors marked. The large blue block indicates the attachment of the blade exciter, and the smaller red blocks indicate the faults.

In utilizing AE sensors to detect internal manufacturing defects under cyclic loading, Mielke et al. [44] studied full-scale wind turbine blades 14.3 m and 31 m long, as shown in Figure 10. They found that while it was straightforward to trace AE signals back to embedded manufacturing faults, the acoustic wave characteristics were inconsistent across different testing phases.

In the field of damage localization, researchers are committed to improving localization accuracy and reducing system complexity. Multiple studies have explored this from different technical paths. Regarding damage source localization methods, Zhao et al. [45] obtained the dispersion relationship of the blade structure through a semi-analytical finite element method and on this basis conducted an in-depth study of the propagation characteristics of AE waves in key structures. They compared five methods for determining the difference in the time of arrival to improve numerical accuracy. The results showed that this method is efficient and reliable and can serve as a technical foundation for building a digital twin of the blade structure.

Another line of research explores the use of π-phase-shifted fiber Bragg gratings (FBGs) as sensing elements, which offer approximately twice the sensitivity of conventional FBGs and overcome several limitations associated with piezoelectric transducers [46]. In tests conducted on small wind turbine blades, these enhanced FBG sensors successfully captured acoustic emission signatures associated with various damage conditions. The resulting feature datasets provide an essential basis for subsequent work on accurate damage localization and discrimination across different severity levels.

There is also research in the industry on AE flaw detection simulation. Jiang et al. [47] used the NREL 5MW wind turbine blade as the object. Through precise flow field simulation and damage simulation, they predicted that the blade is more prone to damage between the 11th and 13th cross-sections and identified three damage types—matrix damage, delamination failure, and fiber damage—and their occurrence sequence. This simulation analysis can effectively guide the optimal placement of AE detection equipment on long flexible blades, enhancing the targeting of monitoring and localization by pre-judging hazardous areas.

In the field of damage identification and classification, advanced signal processing and machine learning algorithms are widely used to distinguish complex damage modes. Wang et al. [48] used cluster analysis and the wavelet packet transform to identify four typical damage modes—matrix damage, fiber fracture, etc.—in tensile experiments of composite laminates and clearly revealed their evolution process.

To overcome the limitations of traditional methods in feature extraction, Samareh-Mousavi et al. [49] and Benzon et al. [50], in monitoring delamination damage, effectively tracked the stable growth of damage by analyzing parameters such as the frequency range and energy of acoustic emission signals, combined with statistical methods, and correlated AE activity with the location of the delamination front.

A further advancement involves leveraging combined AE and strain measurements to enable early-stage failure prediction for wind turbine blades [51]. Building on these multi-modal signals, a fault early-warning model was developed that extracts discriminative acoustic features indicative of incipient damage. This predictive capability allows maintenance actions to be planned proactively, thereby reducing the likelihood of critical failures and effectively extending blade service life.

The advantages of AE technology lie in its high sensitivity and real-time capability. However, challenges include the need for a large number of sensors and susceptibility to environmental noise interference. Current research, by introducing graph neural networks, deep learning, advanced signal processing algorithms, and multi-technology fusion strategies, has significantly improved the accuracy, automation level, and engineering applicability of acoustic emission technology in damage localization, identification, and monitoring of wind turbine blades, laying a solid foundation for promoting the technology from the laboratory to practical application in wind farms.

#### 2.2.2. Passive Acoustics and Active Acoustic Excitation Detection Methods

In addition to AE signals emitted by the material itself, using changes in the acoustic field inside and outside the blade for detection is also an effective method, which can be divided into passive and active acoustic detection methods.

Passive acoustic methods rely on acoustic excitation generated by the environment or operation itself. Solimine et al. [52] proposed installing microphones inside the blade to monitor changes in sound pressure levels caused by natural wind excitation. The preliminary audio signal processing steps used in the study were significantly influenced by speech processing methods. By analyzing the feature space representation of the dataset through Principal Component Analysis (PCA) and K-means clustering, they successfully detected structural and acoustic anomalies experienced by a full-scale wind turbine blade during fatigue testing.

**Figure 11:**
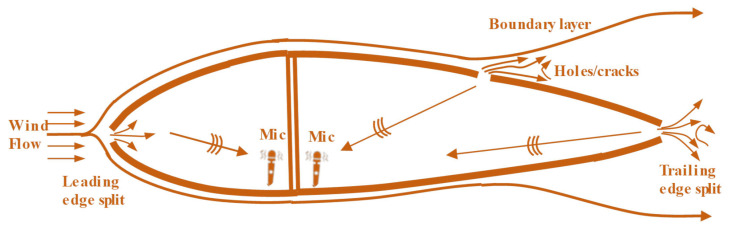
Schematic illustration of the passive-based damage detection technique described by Beale et al. [53].

As shown in Figure 11, Beale et al. [53] placed a speaker inside the blade cavity in their study to actively excite the sound field and measured the acoustic pressure response through an external microphone array. They found that when the blade is damaged, the structural acoustic transfer characteristics change, leading to a significant increase in the acoustic energy measured externally. This method can even detect damage with a length of 5.1 cm.

An alternative acoustic-based strategy employs a microphone array to detect blade anomalies through their radiated sound fields, enabling the identification of cracks or damage with a comparatively simplified sensing and processing workflow [54].

Building upon acoustic diagnostics, subsequent work has incorporated data-driven methods to further enhance detection accuracy. One study analyzed turbine noise by feeding spectrogram representations of acoustic signals, together with rotor-speed information, into a convolutional neural network, achieving a blade surface damage classification accuracy of up to 97.11% [55]. This demonstrates the strong potential of deep-learning-based acoustic analysis for reliable, non-contact blade condition monitoring.

**Figure 12:**
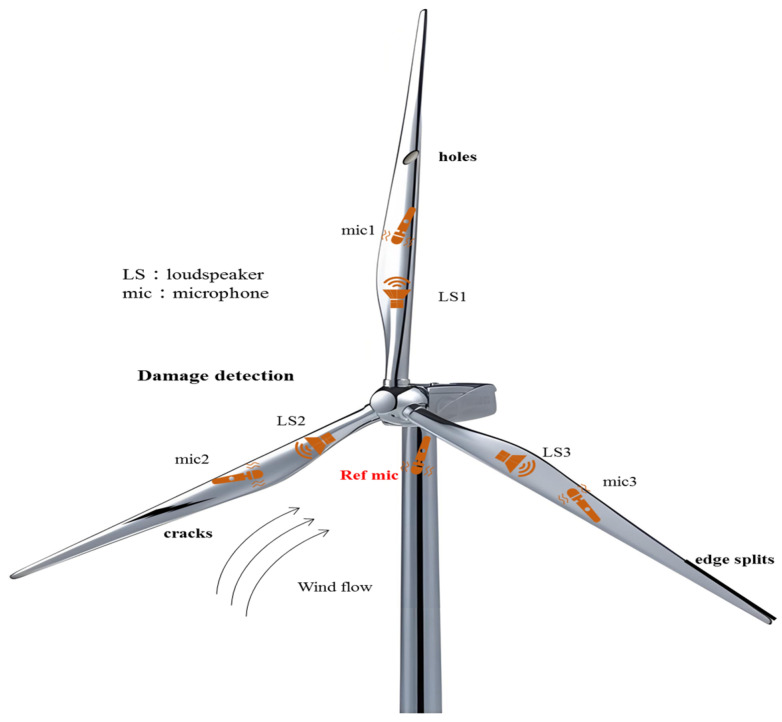
Schematic diagram of active acoustic detection of wind turbine blade damage described by Beale et al. [56].

Active acoustic techniques, illustrated in Figure 12, deploy distributed sensor arrays along the blade surface to capture acoustic emission signals from multiple viewpoints in real time. Such spatially resolved acquisition enhances the accuracy of damage localization and enables effective tracking of damage evolution, making this approach increasingly prevalent in blade health monitoring applications [56].

Active acoustic monitoring has expanded from surface sensing to internally excited interrogation and physics-informed far-field diagnostics, demonstrating strong adaptability for probing complex composite blades. Both internally generated acoustic excitation and aeroacoustic response analysis have been shown to extract defect-related acoustic signatures for early-stage anomaly recognition, validating the feasibility of non-contact, system-level blade defect assessment. These advances highlight the growing consensus that active and aeroacoustic modalities provide a scalable acoustic diagnostics foundation for incipient damage detection and support more proactive blade health monitoring within integrated wind turbine SHM frameworks [57,58].

#### 2.2.3. Summary of Acoustic Testing Methods

In this section, the advantages and disadvantages identified by various researchers in the application of acoustic testing methods in wind turbine blades are shown in Table 3.

In summary, acoustic emission technology can monitor the expansion of material internal damage in real time, offering significant advantages for structural health monitoring. However, its practical application faces notable bottlenecks. The primary challenge is its susceptibility to environmental noise, as wind, waves (in offshore settings), and mechanical vibrations from turbine operation can mask or distort genuine damage-related signals, necessitating complex denoising algorithms. Furthermore, signal attenuation in large composite blades limits the effective sensing range, requiring dense sensor arrays that increase system complexity and cost. Despite these challenges, passive acoustic technology has broad application prospects in structural health monitoring, while active acoustic technology shows promise in blade monitoring, vibration control, and noise reduction. In the future, acoustic testing technology, combined with advanced signal processing and artificial intelligence, is expected to overcome these limitations and achieve more efficient and accurate blade health management, providing strong support for the sustainable development of the wind power industry.

### 2.3. Vibration Testing Methods

Vibration monitoring technology is another commonly used method for fault detection in wind turbine blades. By arranging vibration sensors (such as accelerometers) on blades, vibration signals generated during blade rotation can be detected in real time [59]. Vibration signals contain various types of information about blade structure, load, and external environment. By analyzing the characteristics of these vibration signals, the damage status of wind turbine blades can be identified [59]. This method infers the health status of blades by monitoring vibration signals and analyzing their characteristic changes.

#### 2.3.1. Modal Analysis and Frequency Response Function Method

This method identifies modal parameters such as natural frequencies, damping ratios, and mode shapes by measuring the response of blades under known excitation (e.g., an impact hammer or shaker) and obtaining their frequency response function [60]. When blade damage occurs, local stiffness decreases, leading to changes in natural frequencies and abnormalities in mode shapes.

Researchers have made many attempts in active excitation. For example, Fremmelev et al. [61] sequentially introduced various types of artificial damage on a 52 m wind turbine blade and conducted fatigue tests for each damage type. During fatigue testing, they used features based on active vibration data to detect the initiation and progression of damage in wind turbine blades, showing a good correlation between observed damage progression and calculated damage index changes.

Active vibration can also be used for early identification of structural defects in blades. Fremmelev et al. [61] conducted monitoring on a 52 m full-scale blade. They found that low-order modes were insensitive to minor damage and thus adopted an active vibration system consisting of exciters and distributed accelerometers. Through frequency-domain feature extraction and outlier detection, they successfully tracked the initiation and progression of damage during fatigue testing. In addition, Tcherniak et al. [62] designed an active vibration monitoring system combining sensor arrays and data processing algorithms capable of real-time detection of blade cracks, edge cracking, and other defects.

Video- and vision-assisted vibration analysis has attracted sustained research interest, with multiple studies confirming the feasibility of extracting blade damage indicators from complex vibration behaviors [63,64]. Time–frequency-based analytics have been explored to reveal characteristic damage signatures embedded in non-stationary blade vibration responses, with simulations showing strong capability in distinguishing abnormal vibration patterns, supporting damage-related feature discovery for intelligent SHM pipelines [63,64]. Signal-based spatial damage assessment has further incorporated model-informed dynamic features, including acceleration responses acquired from multi-sensor configurations on a tower along with SCADA operational context, demonstrating the ability to infer blade damage locations using curvature-derived mode shape deviations in numerical environments [64]. Nevertheless, classical frequency-domain health indicators are broadly acknowledged to exhibit limited sensitivity to early-stage local damage and to be strongly modulated by environmental variability, such as temperature, which can obscure subtle structural changes induced by incipient defects [64].

#### 2.3.2. Operational Modal Analysis Method

Operational Modal Analysis (OMA), as a non-destructive testing technology, has also been applied in wind turbine blade damage monitoring. OMA technology only requires measuring the response of structures under environmental excitation, without measuring input forces, making it very suitable for monitoring in-service wind turbines. The advantage of OMA is that it can reflect the dynamic characteristics of structures under actual operating conditions.

Field monitoring of an onshore 2.0 MW wind turbine over a period exceeding one year demonstrated the practical effectiveness of integrated structural health monitoring systems [65]. By establishing regression models to mitigate the influence of environmental and operational variability, the study showed that the system can reliably detect damage across blades, foundations, and towers.

In a complementary approach, co-simulation using NREL FAST and ANSYS allowed the identification and localization of longitudinal cracks in the upper sections of a blade using only acceleration responses under wind excitation, combined with a frequency-domain decomposition algorithm [66]. This method highlights a feasible pathway toward low-cost, high-fidelity monitoring of wind turbine structures.

For floating offshore wind turbines, operational modal analysis based on numerically simulated sensor signals enabled the extraction of modal parameters for both towers and blades [67]. The analysis indicated that curvature mode shapes are particularly sensitive to damage, providing the most reliable indicators of both location and severity, thus offering valuable guidance for effective offshore blade monitoring strategies. With advancements in vibration sensor technology, real-time vibration monitoring of wind turbine blades has become a feasible operation. By arranging vibration sensor arrays on blade surfaces, comprehensive vibration data during blade operation can be collected. Rezamand et al. [68] combined data analysis techniques such as wavelet transform and time–frequency analysis to extract damage-related feature parameters from vibration signals, enabling real-time assessment of blade health status.

Advanced technologies have been integrated into data analysis. For example, research by Rangel-Rodriguez et al. [24] and Ogaili et al. [69] highlighted the powerful capability of machine learning in damage classification and severity assessment. The former achieved over 99.5% classification accuracy for four states—healthy, minor, moderate, and severe cracks—by combining ANOVA feature selection and the K-nearest neighbors (KNN) algorithm. The latter demonstrated that combining the ReliefF feature selection algorithm with the KNN classifier can enable the effective identification of cracks at different blade locations, with an overall accuracy of 97%.

#### 2.3.3. Summary of Vibration Testing Methods

In this section, the advantages and disadvantages identified by various researchers in the application of vibration testing methods in wind turbine blades are shown in Table 4.

In summary, significant progress has been made in vibration signal analysis for damage detection in wind turbine blades. From traditional time–frequency analysis and statistical methods, it has developed to comprehensive analysis of vibration signals using multi-sensor fusion and intelligent algorithms. However, a key technical bottleneck for vibration-based methods remains the strong dependency on excitation frequency selection. Low-order modes are often insensitive to minor, localized damage, while high-frequency responses are more susceptible to attenuation and noise. Additionally, vibrational characteristics are significantly influenced by environmental and operational conditions (e.g., temperature, wind speed, and rotor speed), making it challenging to isolate damage-induced changes from normal operational variability. In the future, vibration analysis will combine with other detection methods to achieve multi-modal fusion innovation and introduce advanced machine learning and deep learning technologies to improve detection sensitivity and robustness, thereby addressing these longstanding limitations.

### 2.4. Ultrasonic Testing Methods

Ultrasonic testing utilizes the propagation characteristics of high-frequency sound waves in materials to detect internal defects, being particularly sensitive to damage such as delamination and porosity. Ultrasonic testing technology, with its high resolution and deep detection capability, is widely used for detecting internal defects in wind turbine blades, especially showing unique advantages in detecting resin stripping, interlaminar cracks, and bubbles [70].

**Figure 13:**
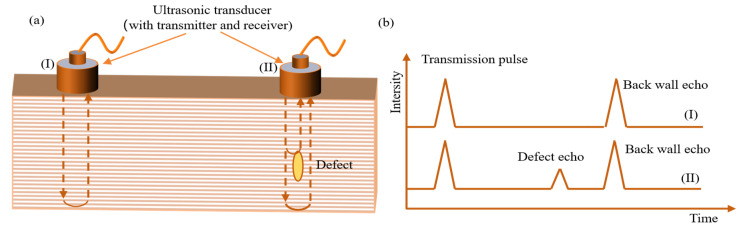
Principal of the pulse–echo technique [70]. (**a**) Ultrasonic testing device. (**b**) Detecting waveforms.

**Figure 14:**
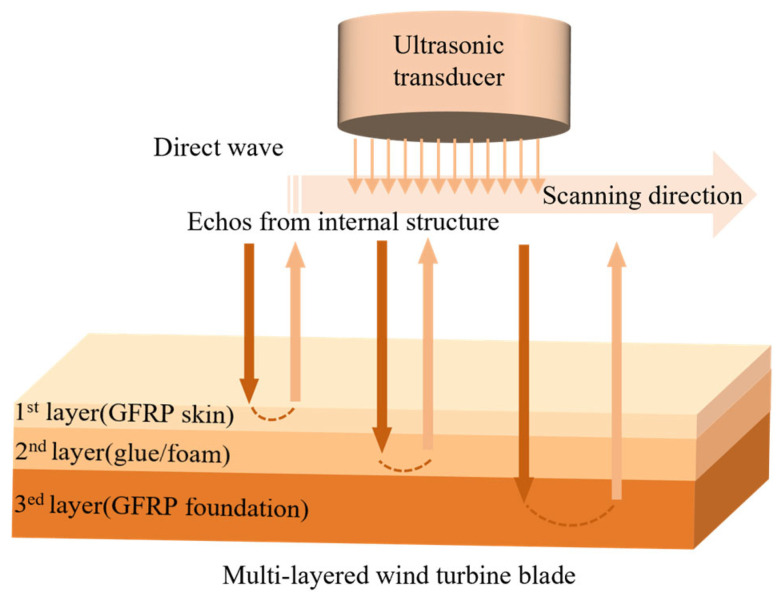
Pulse–echo technique mechanism used for the investigation of wind turbine blades [70].

An ultrasonic detection device is shown in Figure 13a. The ultrasonic sensor contains a transmitter and a receiver. The transmitter sends a pulse wave, and the receiver is responsible for receiving the return wave. If the material is damaged, two or more return waves will appear. The internal defects in the material can be analyzed based on the characteristics of these return waves, as shown in Figure 13b. Figure 14 shows a schematic diagram of using a movable ultrasonic testing device to detect internal defects in wind turbine blades.

#### 2.4.1. Ultrasonic Guided-Wave Testing Technology

In traditional ultrasonic testing, single-frequency sound waves are used to detect internal defects in blades. However, with the increasing complexity of wind turbine blade structures, single-frequency ultrasonic signals may not comprehensively detect all types of damage, especially those that can propagate long distances in plate-like or shell-like structures, and thus rapid detection over large areas may not be possible [71].

In recent years, research based on high-frequency ultrasound has made significant progress, mainly reflected in the following aspects. To verify the feasibility of actuator-sensor networks, Yang et al. [71] successfully localized blade damage in a laboratory environment through guided-wave pitch–catch SHM technology.

Regarding the feasibility of using guided waves for delamination detection and localization, Muñoz et al. [72] successfully detected delamination defects at a distance of 4 m from a sensor by using the wavelet transform for signal denoising and adopting a correlation-based pattern recognition method for two real blades.

There is also research on using guided waves to evaluate mud accumulation on blade surfaces. Márquez et al. [22] used Macro-Fiber Composite (MFC) sensor-based and neural network technology for structural health monitoring, with different grades of mud accumulation used to validate experimental results, acquiring signals at different excitation frequencies. Experimental results showed that at a 25 kHz frequency, the neural network classification accuracy was 100%, indicating the high accuracy of the method.

Guided-wave ultrasonics are valued for long-range propagation and large-area coverage, enabling scalable blade inspection with sparse sensors [73,74]. Studies have confirmed that guided waves can traverse plate-like composite blade structures, allowing wide-region interrogation from limited sensing points [73]. Hybrid configurations that pair curvature-conformal piezoelectric carriers with laser ultrasonic excitation further extend feasibility to non-planar blade surfaces, collectively providing a flexible ultrasonic sensing pathway for localized defect detection in composite blades [74].

Ultrasonic testing for blade monitoring has increasingly migrated toward robotic and data processing-enhanced architectures. A UAV-borne, negative-pressure adsorption crawler integrated with ultrasonic sensors enabled non-stop online internal defect scanning, reporting 100% detection capability in field validation [75]. Anomaly recognition using only healthy-state ultrasonic baselines was achieved through a signal processing chain combining wavelet denoising, PCA, and novelty detection, offering a solution for one-class classification scenarios [76]. Guided-wave phase-velocity shifts were further incorporated as physics-informed indicators for estimating defect location and size, yielding relative errors of 2.7% (simulation) and 10% (experiment) [77]. Sparse-domain decomposition via an improved matching pursuit framework enhanced time-of-flight estimation and detection of weak, hidden defect reflections [78].

#### 2.4.2. Phased Array Ultrasonic Technology

**Figure 15:**
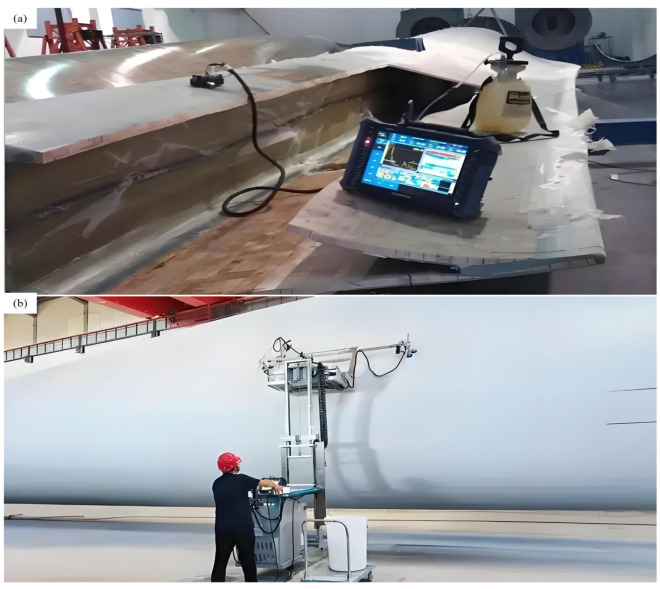
Ultrasonic phased array flaw detector. (**a**) Blade phased array detector. (**b**) Automatic scanner based on phased array. (Source: NISYO-GOYO).

Phased array ultrasonic technology electronically controls the excitation timing of individual crystals in array probes, achieving beam steering and focusing without moving the probe [79]. Figure 15 shows an ultrasonic phased array flaw detector. High-frequency ultrasound can detect micro-cracks and surface-layer damage, while phased array technology can achieve rapid scanning of large areas through multi-probe arrays. Phased array technology can provide two-dimensional or even three-dimensional defect imaging, greatly improving detection accuracy and speed [79].

Phased array ultrasonic technology can accurately locate artificial defects and impact damage in carbon fiber composite laminates. Caminero et al. [79] quantified damage area through research on C-scan images of phased array ultrasound, providing a detection method with higher resolution and reliability than traditional ultrasonic testing.

In recent years, ultrasonic phased array technology has begun to be widely used in defect detection for wind turbine blades. Zhang et al. [80] analyzed detection methods for common defects in the production stage, emphasizing the importance of ultrasonic phased array techniques in early defect identification. This research shows that ultrasonic phased array technology can effectively identify minor defects in blades, preventing potential mechanical failures.

Additionally, for early detection of minor damage in blades, Sun et al. [81] proposed an ultrasonic phased array element layout method based on a Fermat spiral array, significantly improving the array fill factor and reducing grating and side-lobe effects, thereby achieving high-energy excitation and precise imaging. This innovative layout provides higher spatial resolution and detection accuracy for ultrasonic testing of wind turbine blades.

#### 2.4.3. Summary of Ultrasonic Testing Methods

In this section, the advantages and disadvantages identified by various researchers in the application of ultrasonic testing methods in wind turbine blades are shown in Table 5.

In summary, ultrasonic testing of wind turbine blades is rapidly transitioning from traditional manual inspection to a new paradigm integrating intelligence, automation, and full-field structural health monitoring. However, despite its high sensitivity to internal defects, ultrasonic testing faces significant bottlenecks when applied to complex blade structures. Signal attenuation and mode conversion occur as waves interact with geometric features such as stiffeners, curvature, and sandwich cores, complicating data interpretation. For in-service blades, the need for a couplant and the difficulty of deploying contact transducers on large, curved surfaces remain practical challenges, particularly in offshore environments. In the future, further deep integration with data-driven algorithms such as deep learning and digital twins will certainly lay the foundation for overcoming these limitations and achieving autonomous, predictive, and high-precision integrity management of the next generation of wind energy systems.

### 2.5. Thermal Imaging Testing Methods

Thermal imaging testing methods use infrared cameras to measure the infrared radiation from object surfaces to create temperature distribution maps, thereby revealing surface or near-surface defects. This technology has demonstrated powerful advantages in surface defect monitoring of wind turbine blades, especially suitable for real-time monitoring and dynamic analysis [82].

#### 2.5.1. Passive and Active Thermal Imaging Technology

Passive thermography directly uses solar radiation or the equipment’s own heat generation as the heat source to monitor the temperature distribution of blades. For example, sunlight heating of blades may cause areas with internal defects (such as delamination) to exhibit different surface temperatures compared to defect-free areas. Sanati et al. [82] validated the effectiveness of thermal imaging technology through experiments on severely damaged blade sections and small “plates” with known defects. Passive thermography combined with “step heating phase and amplitude thermography” methods can improve image quality and eliminate false alarms caused by environmental reflections and surface dirt. The research also showed that morning and noon are the best times for passive thermography to maximize defect detection.

**Figure 16:**
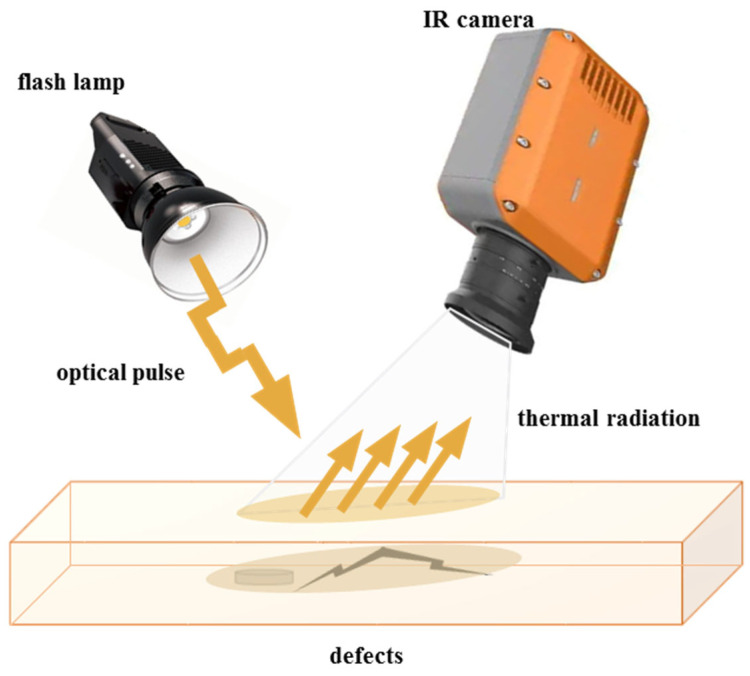
Principle of active thermal imaging detection method.

Active thermal imaging technology, as shown in Figure 16, uses external heat sources to actively excite blades. By analyzing surface temperature differences formed due to defect obstruction during internal heat conduction, internal defects can be detected. Pulse thermography and lock-in thermography are two representative methods.

Pulse thermography uses short-duration, high-energy pulses for heating, featuring rapid and comprehensive characteristics, and is widely used for preliminary screening of various defects. For example, research by He et al. [83] and Tao et al. [84] showed that pulse thermography can effectively detect bonding defects in GFRP blades with an efficiency far higher than traditional ultrasonic methods. Doroshtnasir et al. [85] further studied the capability of thermal imaging technology for detecting potential defects in rotating blades in the field, demonstrating the potential of this technology in long-distance monitoring. Jensen et al. [86] used long-pulse thermography technology to successfully detect potential early-stage defects in curved and coated structures, providing a technical basis for early warning.

Lock-in thermography is also a type of active thermography that uses periodically modulated thermal waves for excitation. By extracting the amplitude and phase information of thermal wave signals, it has better detection capability for deep defects. Jensen et al. [86] applied it to specimens with strong curvatures and coatings, successfully detecting subsurface defects with depth-to-diameter ratios of up to 1.04, demonstrating its detection potential in complex parts such as simulated blade leading edges. In addition, Manohar et al. [87] successfully combined lock-in thermography with multivariate outlier analysis for delamination detection in a 9 m long blade, effectively suppressing the “blind frequency” effect and improving defect contrast.

Additionally, long-pulse thermography, as another active excitation method, is suitable for thicker materials or materials with low thermal conductivity that require longer heating times.

#### 2.5.2. Advanced Thermal Imaging Methods Combining Optical Excitation and Remote Scanning

**Figure 17:**
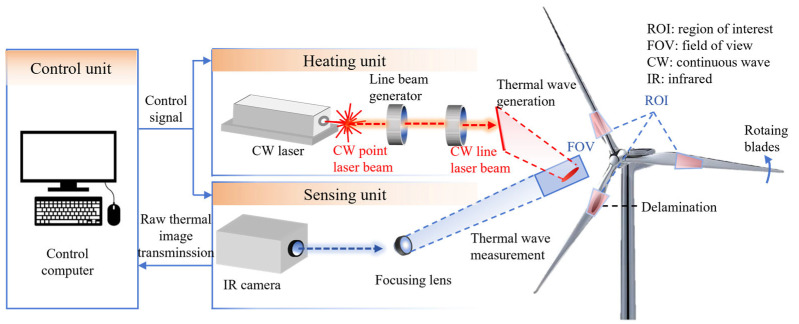
Schematic representation of the proposed continuous-wave line laser thermography [88].

To achieve non-contact, long-distance precise detection of moving blades, technologies combining optical excitation with thermal imaging have developed rapidly. These technologies use lasers as precise heat sources and possess advantages of concentrated energy and good directivity. Hwang et al. [88,89] conducted a series of studies in this field. They developed a continuous-wave line laser scanning thermography system that can detect rotating blades from distances of up to 10 m and successfully identify internal delamination via the research method shown in Figure 17. The key to this method lies in its ability to generate and analyze laser-induced thermal wave propagation patterns, thereby achieving instantaneous detection and quantification of defects without relying on baseline data.

Another optical excitation method is flash-lamp pulse thermography, which, although not laser-based, also belongs to non-contact optical excitation. Zhang et al. [90], when using step-heating thermography technology, significantly improved the depth detection accuracy for defects in large-blade spar caps by establishing a three-dimensional heat conduction model, overcoming the limitations of traditional one-dimensional models that ignore lateral heat diffusion.

Pulse thermography is a type of active thermography that heats the object surface with short-duration, high-energy pulses. Yang et al. [91] combined induction heating with thermography, introducing thermal wave radar analysis technology based on cross-correlation pulse compression and matched filtering. This technology can significantly improve signal-to-noise ratio and defect shape recognition capability. Experimental results showed that compared to traditional eddy current pulse thermography and eddy current pulse phase thermography, thermal wave radar (TWR) can more effectively suppress non-uniform heating effects and fiber fabric structure interference in carbon fiber composites, thus more clearly revealing internal delamination defects.

#### 2.5.3. Surface and Near-Surface Detection Technology Combining Eddy Current Effects

**Figure 18:**
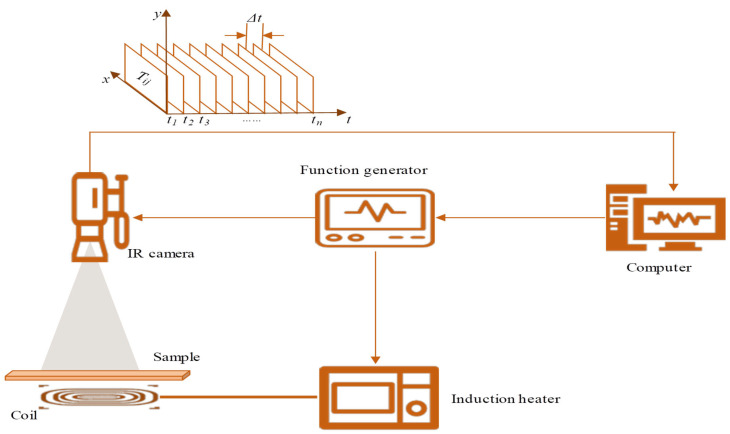
Diagram of an eddy current pulsed thermography system [93].

For surface and near-surface defect detection of carbon fiber-reinforced composites, the main material of wind turbine blades, pulsed eddy current thermography shows unique advantages. The principle of this technology is shown in Figure 18, using eddy current effects for heating. Defects disturb the uniform distribution of eddy currents, thereby changing the surface thermal field. Cheng et al. [92] showed that this method can not only detect surface cracks in carbon fiber composites but also characterize the anisotropic conductivity of materials by analyzing surface heating patterns.

Liang et al. [93] used this technology to detect low-energy impact damage and innovatively adopted a multi-resolution statistical method combining the wavelet transform and Principal Component Analysis to extract weak defect features from original thermal images, greatly improving the detection capability for small-sized damage.

Regardless of the excitation method used, advanced data processing and intelligent algorithms are key to improving detection efficiency. In recent years, machine learning and deep learning algorithms have been widely introduced. Reta et al. [94] used K-means clustering for defect region segmentation and successfully achieved automatic classification of various subsurface defects using a naive Bayesian classifier. Chon et al. [95] went further by generating and preprocessing synthetic thermal imaging data to train deep neural network models, effectively solving the problems of scarce real defect data and class imbalance, significantly improving the model’s generalization performance and recognition accuracy on real data.

#### 2.5.4. Summary of Thermal Imaging Testing Methods

In this section, the advantages and disadvantages identified by various researchers in the application of thermal imaging technology in wind turbine blades are shown in Table 6.

In summary, thermal imaging technology has broad application prospects in damage detection in wind turbine blades. Its advantages lie in non-contact, remote, and rapid detection, especially suitable for monitoring in complex environments and large-area blades. However, several technical bottlenecks must be addressed to realize its full potential. The main limitation is its shallow penetration depth, which restricts detection to near-surface defects. In field applications, results are heavily influenced by environmental factors such as ambient temperature, wind speed (which cools the surface), and solar loading, which can create false indications. In the future, combined with multi-modal detection technology, intelligent algorithms, and UAV platforms, the detection accuracy and efficiency of thermal imaging will be further improved. Solving these technical challenges—such as environmental interference and geometric distortion—will be key to promoting the widespread application of thermal imaging technology. As the technology continues to mature, thermal imaging is expected to become an important tool for health monitoring of wind turbine blades, providing solid technical support for the sustainable development of the wind power industry.

### 2.6. Performance Comparison, Challenges and Limitations: Analysis of Various NDT Methods

After systematically reviewing traditional technologies, it can be seen that different NDT technologies have their own characteristics in terms of detection capability, cost, automation level, etc., due to their different physical principles and application methods. Table 7 provides a systematic performance comparison and deeply analyzes the challenges and limitations in current research, which is crucial for correct technology selection and future development.

In conclusion, the optimal approach for comprehensive blade health management, particularly in demanding offshore conditions, lies in hybrid strategies. These involve synergistically combining continuous, global monitoring methods with periodic, high-precision inspections using multi-sensor robotic platforms. This integrated philosophy directly supports the transition toward the intelligent, data-driven frameworks discussed in the following sections.

## 3. Emerging Intelligent Inspection Technologies and Data-Driven Methods

With the rapid development of sensing technology, computational capabilities, and artificial intelligence algorithms, the field of NDT for wind turbine blades is undergoing a profound paradigm shift [96]. Data-driven methods and intelligent technology platforms are endowing traditional inspection methods with unprecedented levels of automation, precision, and predictive capabilities [97].

### 3.1. Machine Learning and Deep Learning

#### 3.1.1. Supervised Learning for Fault Classification and Diagnosis

Supervised learning algorithms establish mapping models from input features to specific damage categories or states by using labeled training datasets and are widely applied in blade fault classification [98]. Jiménez et al. [99] proposed a method for dirt detection and diagnosis in wind turbine blades based on ultrasonic guided waves and supervised learning classifiers. They employed multiple supervised learning classifiers to classify signals and identify faults, providing a new solution for the reliability of wind power equipment. Subsequently, they validated the model’s effectiveness under icy conditions on wind turbine blades [100]. Finally, the model was applied to the maintenance and management of wind turbine blades.

Regarding the reliability of wind turbine blade maintenance management, Joshuva et al. [59] utilized histogram features extracted from vibration signals and compared multiple lazy learning algorithms, finding that Locally Weighted Learning achieved the highest accuracy of 93.83% when diagnosing various faults such as blade bending and cracks. In acoustic-based detection, Regan et al. [101] systematically evaluated the performance of logistic regression and support vector machines in binary classification tasks of laboratory-scale blade damage and optimized model performance through Fisher ratio-based feature selection.

For more complex defect patterns, Jiménez et al. [102], when using ultrasonic guided waves to detect blade delamination, compared various classifiers such as decision trees, discriminant analysis, and support vector machines, finding that methods combining nonlinear autoregressive exogenous model feature extraction significantly improved classification accuracy. These studies indicate that selecting appropriate combinations of feature engineering and supervised learning algorithms according to different data characteristics and diagnostic objectives is key to achieving high-precision fault diagnosis.

#### 3.1.2. Unsupervised/Semi-Supervised Learning for Anomaly Detection and Health Representation

In engineering deployment, the acquisition of large labeled blade damage datasets remains cost-prohibitive or impractical. Unsupervised and semi-supervised frameworks address label scarcity by constructing normal-behavior baselines from healthy-state data and detecting deviations as anomalies or damage.

Semi-supervised acoustic diagnostics have also advanced through self-supervised health representation learning, trained exclusively on healthy samples. By learning noise- and environment-robust latent health features via neural networks and performing state identification using kernel density estimation, this approach outperformed traditional methods in both laboratory and field measurements [103].

A field implementation using the unsupervised PCA multi-model auto-regressive strategy demonstrated low false alarm rates—4%, 1%, and 0%—for 15 cm, 30 cm, and 45 cm blade damage cases, respectively, when relying on a single vibration sensor under varying operational and environmental conditions [98].

#### 3.1.3. Neural Networks in Image Defect Recognition

**Figure 19:**
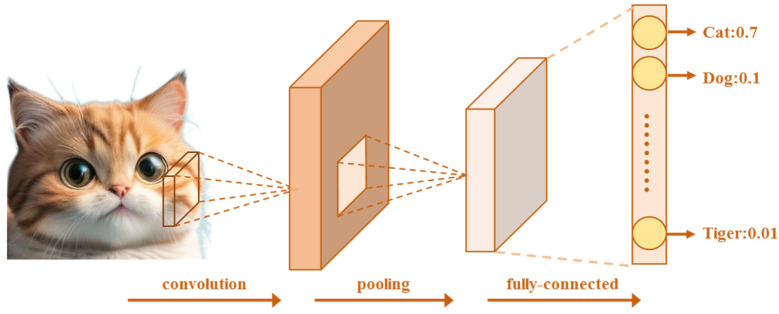
Schematic of Convolutional Neural Network [96].

In the field of ultrasonic testing, CNNs have been successfully applied to defect detection in wind turbine blades [96], with the principle shown in Figure 19. Mendikute et al. [96] pointed out that applying CNNs to ultrasonic testing data can achieve automated identification and classification of internal defects in wind turbine blades, which is crucial for ensuring the safe operation of wind turbine blades.

To address the problem of detecting different types of artificial damage on operating wind turbine blades, Movsessian et al. [25] proposed a method based on artificial neural networks. By establishing a nonlinear relationship between damage-sensitive features in healthy states and a novelty index calculated using Mahalanobis distances, they effectively overcame the impact of environmental and operational variable changes.

YOLO (You Only Look Once) series models are widely used due to their good balance between speed and accuracy [104]. For detecting small-sized defects such as cracks and oil stains, Qiu et al. [105] proposed a YOLO-based small target detection method that enhances detection through multi-scale feature pyramids, achieving a mean average precision of 91.3% on large datasets.

**Figure 20:**
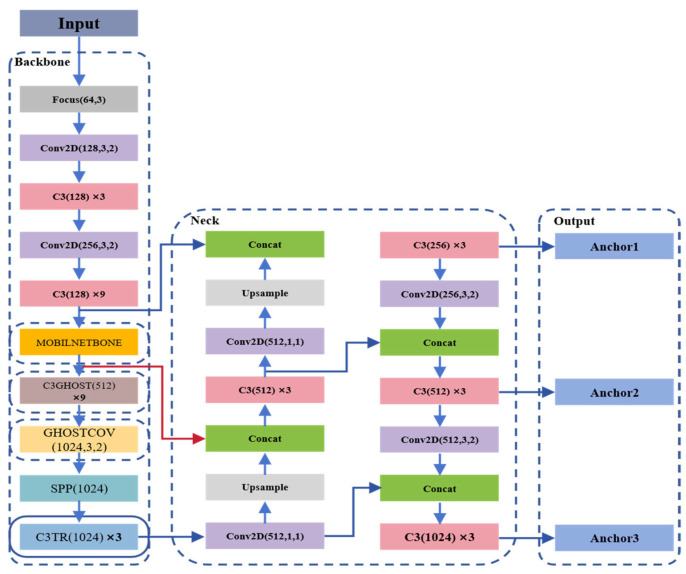
Structure of MI-YOLO developed by Zhu et al. [106].

Regarding the detection capability for light-colored and low-resolution cracks, Zhu et al. [106] proposed a wind turbine blade crack detection method named Multivariate Information YOLO (MI-YOLO), as shown in Figure 20. Hang et al. [13] tested the detection performance of the proposed method using blade images with cracks captured by UAVs. Experimental results showed that MI-YOLO can effectively achieve blade fault diagnosis. To optimize model deployment on mobile devices or embedded systems, Zhang et al. [107] proposed an attention mechanism-based MobileNetv1-YOLOv4 model, significantly reducing computational complexity while maintaining high accuracy.

These deep learning methods not only have high accuracy but also lay the technical foundation for automated, high-frequency blade inspection in large-scale wind farms.

Furthermore, YOLO series models have also been applied to ultrasonic testing and thermal imaging for wind turbine blade detection, validating the detection effectiveness of this model combined with the aforementioned technologies [107,108].

In summary, machine learning and deep learning are driving the paradigm shift in wind turbine blade non-destructive testing towards data-driven intelligent approaches. Supervised learning demonstrates high accuracy in various fault classification tasks, while unsupervised and semi-supervised learning provide effective paths for anomaly detection under label scarcity. Deep learning models represented by CNNs and the YOLO series, with their powerful feature extraction and defect recognition capabilities in image and ultrasonic data, are becoming the core technological foundation for achieving automated, high-precision blade health diagnosis and inspection.

### 3.2. Advanced Signal Processing and Feature Extraction

#### 3.2.1. Time–Frequency Analysis Based on Wavelet Transform

Non-stationary acoustic or vibration signals often contain rich damage information. The wavelet transform, as an excellent time–frequency analysis tool, can analyze signals in both time and frequency domains simultaneously, making it very suitable for processing such transient signals [109]. Beale et al. [110] proposed an adaptive wavelet packet denoising algorithm that significantly improved the signal-to-noise ratio of active acoustic damage detection by optimizing noise estimation and threshold selection strategies, increasing damage detection performance by up to 60%.

Chen et al. [111] used the wavelet packet energy ratio to characterize the differences between acoustic pulses from intact and cracked blades and used it as an input feature for an improved incremental support vector data description model, achieving efficient acoustic damage detection. Xu et al. [112] developed a waveform-based feature extraction method based on wavelet packet decomposition (WPD) to capture information in raw acoustic emission signals. Without signal preprocessing, cluster analysis was performed based on the extracted features to achieve damage pattern recognition and singular signal detection. This method demonstrated good robustness under noise interference.

#### 3.2.2. Principal Component Analysis and Nonlinear Energy Feature Extraction

For high-dimensional blade condition data, dimensionality reduction and feature selection are essential for improving model efficiency. PCA, a classical linear dimensionality reduction technique, remains widely adopted in blade defect analysis [68,90,92,102]. A hybrid architecture combining recursive PCA, a GRNN ensemble with single imputation, and wavelet-based PDFs improved detection reliability, enabled earlier fault warnings, and reduced false alarms [68]. Artifact suppression and defect recognition latency were further improved through PCA variants incorporating contrast-enhancement factors and additional contrast-weighting terms, achieving faster defect identification [90]. A multi-resolution feature extraction pipeline integrating the wavelet transform with PCA enhanced the discriminability of small-impact-induced defects in CFRP by strengthening thermal-image feature representation [93]. Comparative evaluations for surface contamination classification also showed that PCA provides stronger downstream classification performance than autoregressive models [102].

However, as blade damage progression often presents nonlinear dynamic behavior, nonlinear feature learning methods exhibit distinct advantages beyond linear subspace projections.

#### 3.2.3. SCADA Data Mining and Digital Twin Technology

The SCADA system of wind turbines streams high-frequency, multivariate operational parameters that encode substantial information on structural and component-level health. SCADA-driven data mining enables low-cost, non-intrusive, and scalable early fault warning for blade failures [113]. Blade breakage and fracture-related operational signatures have been successfully extracted from SCADA datasets to identify failure events across multiple wind farms, validating the feasibility of SCADA-based blade breakage monitoring at the fleet level [86,87,88,89,113].

To model parameter interdependencies and temporal dynamics, deep generative structures—such as conditional convolutional autoencoder variants and correlation-matrix-based analytics—have been introduced to capture system-wide dynamic shifts via reconstruction or statistical deviation errors [87,88]. Pattern-learning and alarm-motif discovery methods, including IoT-enabled motif and motif-like pattern analysis, expand SCADA diagnostics toward IoT-SCADA alarm pattern recognition for blade fault detection [89].

As an advanced monitoring paradigm, digital twin (DT) technologies build high-fidelity virtual mirrors of physical blades through bidirectional mapping and cyber–physical interaction.

Collectively, SCADA analytics and advanced signal processing frameworks are driving blade NDT from single-source assessment toward globally fused, system-level health monitoring. Regarding future developments, SHM architectures increasingly identify SCADA–DT integration as a pathway to real-time, high-fidelity blade state mirroring and online predictive diagnosis, forming a foundation for intelligent, proactive wind farm O&M frameworks.

### 3.3. Robotic and Automated Inspection Platforms

#### 3.3.1. UAV-Assisted Inspection Systems

UAVs have become revolutionary tools for external visual inspection of wind turbine blades. They can quickly and safely reach positions difficult for human access and carry various sensors, such as high-definition cameras, thermal imagers, and LiDAR systems [114].

For example, Wang et al. proposed a data-driven framework for automatic detection of surface cracks on wind turbine blades based on UAV-captured images, while Márquez et al. [115] proposed an acoustic detection system for wind turbine structural health monitoring based on UAVs. Sun et al. [75] proposed a method for non-stop online detection of internal defects in wind turbine blades using ultrasonic testing systems mounted on UAVs.

Additionally, Galleguillos et al. [116] used UAVs carrying infrared thermal imagers for maintenance inspection of wind turbine blades, verifying the feasibility of thermal imaging in practical inspections. This method not only improves inspection efficiency but also enables remote monitoring of large-area blades, greatly reducing labor costs and safety risks. Yang et al. [31] used UAVs to collect images and combined transfer learning with deep learning models to achieve high-precision recognition of blade surface defects.

**Figure 21:**
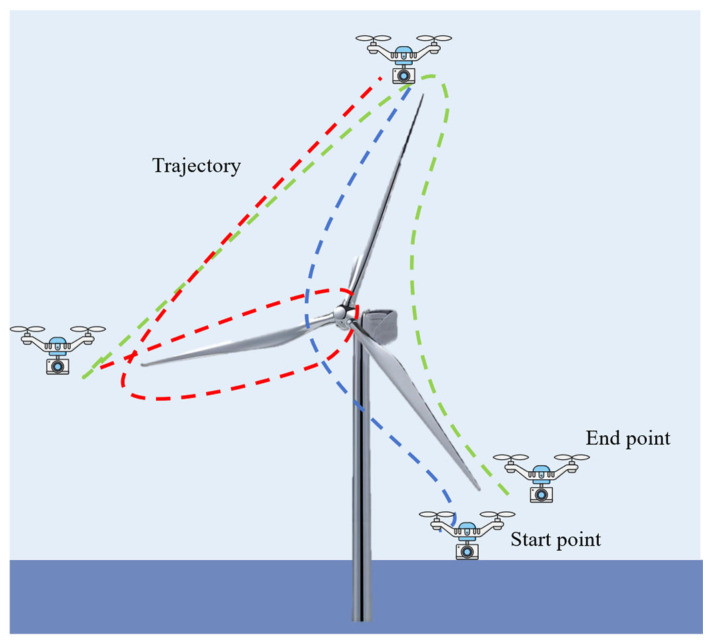
Inspection trajectory of a UAV for WTB and illustration of path planning [118].

UAV technology is developing towards fully autonomous, intelligent diagnosis and is expected to become the standard configuration for regular inspections on wind farms [117,118]. Figure 21 shows an autonomous blade inspection trajectory and path planning for a UAV.

Despite significant progress, current research still has some shortcomings: many advanced algorithms perform well in laboratory environments but their robustness and generalization capabilities need verification when facing complex and changing environmental noise and load conditions in actual wind farms; there is a lack of systematic, standardized comparative evaluation of various inspection technologies, making it difficult for engineers to choose appropriate solutions; research on health monitoring throughout the entire blade lifecycle, especially during the decommissioning stage, is still insufficient.

#### 3.3.2. Crawling Robots and Multi-Sensor Information Fusion

**Figure 22:**
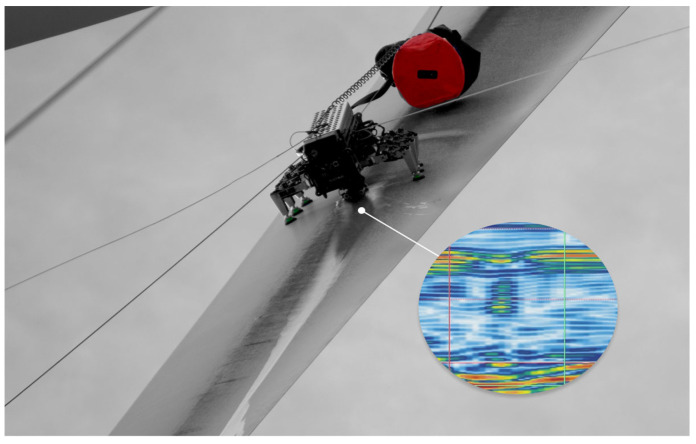
Climbing robots for WTB [119]. (Source: BladeBug).

For internal or contact-based inspection tasks, crawling robotic (Figure 22) platforms provide unique adaptability for accessing confined or complex blade geometries [118,119,120,121,122,123]. Reviews on offshore wind turbine inspection technologies highlight that crawling, underwater, and other mobile robotic systems can deploy multi-sensor payloads—including optical, infrared, and X-ray modules—to achieve high-resolution, close-range assessment of blades and towers [118,119]. Robotic implementations have further demonstrated capabilities for internal blade inspection using micro-/nano-scale X-ray tomography [120], automated quality inspection during blade manufacturing through autonomous manipulators [121], and magnetic-adhesion or levitation-based crawling designs suited for curved composite surfaces [122]. Integrated robotic systems combining inspection, maintenance, and repair functionalities signal a clear trend toward multi-functional, automation-ready platforms for blade health management [123].

Intelligent inspection platforms, including UAVs and climbing robots, have demonstrated considerable engineering effectiveness in practical wind farm O&M applications. Recent studies [124,125,126] indicate that these technologies can substantially reduce O&M costs in offshore wind farms while improving asset availability. In addition, several studies [127,128,129] provide technical validation for large-scale field deployment. Collectively, these cases substantiate the engineering feasibility and economic benefits of emerging intelligent inspection platforms in real-world wind energy applications.

In summary, UAVs and crawling robots have been extensively applied in wind turbine blade inspection. However, how to economically and efficiently integrate high-performance monitoring systems into existing and new wind turbines remains a practical challenge for industrialization. The future trend is to develop highly integrated robotic platforms that collaboratively process data from various sensors such as vision, acoustics, and thermal imaging through multi-sensor information fusion technology to overcome the limitations of single sensing modes and thus obtain more comprehensive and reliable damage diagnosis results in complex field environments.

To provide a clear overview and facilitate comparison, Table 8 summarizes the key emerging intelligent inspection technologies and data-driven methods discussed in this section, highlighting their main applications, strengths, and limitations.

## 4. Challenges and Research Trends in Wind Turbine Blade Inspection Technology

### 4.1. Common Challenges Faced by Current Technologies

Despite significant progress, NDT technologies for wind turbine blades still face a series of common challenges in practical engineering applications.

1. **Interference from Environmental and Operational Conditions:** This is a common problem for various methods such as vibration and acoustic techniques. Variations in wind speed, temperature, humidity, and electromagnetic noise in the wind turbine operating environment can mask or confuse signal changes caused by damage. Effectively separating environmental/operational variations from structural damage variations is key to achieving accurate in-service diagnosis.
2. **Signal Attenuation and the Impact of Complex Structures:** For methods like acoustic emission and ultrasonic guided waves, signals undergo severe attenuation and distortion during propagation in composite materials due to material damping, scattering, and geometric complexity (e.g., stiffeners and curvature), posing significant difficulties for long-distance damage detection and precise localization [120].
3. **Damage Quantification and Early Diagnosis:** Most techniques can detect the presence of damage, but precisely quantifying the size, depth, and severity of damage remains challenging. Furthermore, achieving reliable early warning at the very initial stage of damage initiation (i.e., the “incipient stage”) is crucial for preventive maintenance, but the sensitivity of existing technologies is often insufficient.
4. **Transfer from Laboratory to Engineering Field:** Many advanced technologies (e.g., complex deep learning models and multi-sensor fusion systems) perform excellently in controlled laboratory environments but face severe tests of their reliability, economy, and maintainability when deployed in actual wind farms, which are cost-sensitive, environmentally harsh, and have limited O&M resources. The robustness of algorithms, computational efficiency, and long-term stability of systems require further validation.
5. **Data and Knowledge Barriers:** Data-driven methods urgently require large amounts of high-quality, labeled damage data, which are difficult to obtain in practical engineering. Furthermore, data sharing barriers between different wind farms and turbine models limit model generalization capabilities. How to establish effective diagnostic models using small-sample, cross-domain data is an important future research direction.

### 4.2. Future Research Trends

Based on an in-depth analysis of the current status and challenges in the field of NDT and SHM for wind turbine blades, future research will no longer be limited to innovation in single technologies but will tend towards building a multi-technology integrated, intelligent, and systematic health management ecosystem. The following directions represent the core trends in the future development of this field.

#### 4.2.1. Fusion of Multi-Modal Sensing and Hybrid Inspection Methods

Single non-destructive testing techniques are often only sensitive to specific types or locations of damage, having inherent detection blind spots. In the future, the fusion of multi-modal sensing and hybrid inspection methods will become an inevitable trend. By synergistically using various sensors, such as visual, acoustic, vibration, thermal imaging, and ultrasonic sensors, complementary information and cross-validation can be achieved, enabling more comprehensive and reliable diagnosis of complex damage.

For example, UAVs can simultaneously carry high-definition cameras and thermal imagers, acquiring both surface images and thermal distribution maps of blades in a single flight, thus comprehensively judging surface cracks and near-surface delamination. This integration is reflected not only at the data acquisition level but also at the data fusion algorithm level. Sensor fusion technology can use advanced algorithms to process multi-source heterogeneous data, generating comprehensive diagnostic results that exceed the sum of their parts, effectively suppressing false positives and false negatives [130].

Furthermore, within the digital twin framework, multi-physics simulation models can deeply integrate with multi-modal sensing data, providing unprecedented insights for blade condition assessment and life prediction.

#### 4.2.2. Real-Time Online Monitoring and Edge Intelligent Diagnosis

With the popularization of sensor networks and the advancement of computing technology, the health monitoring of wind turbine blades is transitioning from “regular inspection” and “post-analysis” to “real-time online” and “edge intelligence.” Future monitoring systems will be able to continuously collect data and use edge computing devices deployed on-site at wind farms for real-time or near-real-time data processing and diagnosis. This approach greatly reduces the delay and bandwidth requirements for transmitting data to the cloud, making rapid warning and response to critical blade conditions possible.

The key to realizing this vision lies in developing lightweight intelligent diagnostic algorithms that maintain high accuracy while having low computational complexity to adapt to the resource constraints of edge devices. The self-supervised health representation learning method proposed by Sun et al. [98], which requires only healthy data for training, provides a promising solution for real-time anomaly detection in such resource-constrained scenarios.

#### 4.2.3. Few-Shot Learning, Explainable AI, and Cross-Turbine Transfer Learning

Although deep learning has achieved great success, its in-depth application in engineering fields is facing data and trust bottlenecks. Future research will pay more attention to:

1. **Few-Shot Learning:** Addressing the problem of scarce specific damage samples in actual wind farms, research will focus on how to use technologies such as Generative Adversarial Networks (GANs) and meta-learning to generate or learn effective damage features, achieving diagnostic capabilities of “learning from few examples”.
2. **Explainable Artificial Intelligence:** Complex “black box” models find it difficult to gain engineers’ complete trust. Developing explainable AI models that can reveal the decision basis for damage diagnosis (for example, indicating which area of an image led to crack classification) is crucial for promoting the adoption of AI technology in safety-critical fields.
3. **Cross-Turbine Transfer Learning:** To overcome data barriers, knowledge learned from one wind farm or turbine model can be quickly adapted to a new wind farm or new blade model with scarce data through transfer learning technology, which will significantly reduce model deployment costs and improve generalization capabilities.

#### 4.2.4. Standardization and Certification of Structural Health Monitoring Systems

Currently, there are numerous SHM technologies for wind turbine blades, but there is a lack of unified performance evaluation standards, data interface specifications, and system certification processes, which seriously hinders their large-scale commercial application [131]. An important future task is to promote the standardization of SHM systems. This includes formulating standard definitions of damage-sensitive features, data formats, communication protocols, and system reliability verification procedures.

Simultaneously, the industry needs to establish corresponding certification systems to evaluate and certify the detection accuracy, reliability, and durability of SHM systems, providing confidence and guarantees for wind farm owners and insurance companies to adopt these technologies [132]. Standardization is a key step for SHM technology to move from the laboratory to the mass market.

#### 4.2.5. Full Lifecycle Management and Sustainability of Blades

As the first batch of large-scale installed wind turbines gradually reach their design life, the “retirement wave” of blades is approaching, and issues related to their full lifecycle management and sustainability are becoming increasingly prominent. Future research will no longer be limited to damage detection during the in-service period but will extend to the entire process of blade manufacturing, installation, operation and maintenance, decommissioning, and recycling.

Data generated by SHM systems will be combined with the “digital twin” of the blade to accurately predict the remaining useful life of the blade, providing decision support for life extension operation or timely replacement, thereby maximizing asset value. Furthermore, facing many composite blades about to be decommissioned, it is urgent to develop economical and environmentally friendly recycling and reuse technologies. Non-destructive testing technology can play an important role in this, for example, in quickly assessing the overall condition of blade materials before disassembly to determine the most suitable recycling strategy, thereby promoting true closed-loop and sustainable development in the wind energy industry.

## 5. Conclusions

As critical components of wind energy systems, the health condition of wind turbine blades directly influences the safety, reliability, and economic performance of the wind power industry. This review has systematically examined and critically evaluated the technological progress in NDT and SHM for wind turbine blades. We first synthesized the principles, strengths, and inherent limitations of traditional NDT methods—including vision-based inspection, acoustic techniques, vibration analysis, ultrasound, and infrared thermography—highlighting their applicability to different damage types. Subsequently, the review focused on how data-driven approaches, represented by machine learning and deep learning, are transforming traditional techniques by enabling automated and intelligent damage diagnosis. The analysis further discussed how advanced inspection platforms, such as unmanned aerial vehicles and climbing robots, contribute to higher efficiency, broader coverage, and deeper insight in blade evaluation.

A comprehensive comparative assessment clearly indicates that no single technique is universally applicable. Despite persistent challenges, promising future directions have emerged, including multi-modal technology integration, edge intelligence, advanced data-driven algorithms, and full lifecycle health management frameworks. Looking ahead, a highly intelligent, automated, and fully integrated blade health management system—seamlessly embedded within the broader Industry 4.0 infrastructure—is an achievable vision. Realizing this vision will require sustained interdisciplinary collaboration among materials scientists, mechanical engineers, data scientists, and industry practitioners. Through such collective efforts, continuous technological innovation will further enhance the reliability, economic viability, and sustainability of wind energy as a major clean power source, thereby providing stronger support for the global energy transition.

## Figures and Tables

**Table 1:** Common types of damage in WTBs.

Damage Type	Causes
Surface Damage	High-speed impact from raindrops [10], hail [11], sand particles [12], etc.; manufacturing defects [13]
Internal Damage	Improper manufacturing processes or in-service loads (e.g., impact, fatigue) leading to decreased interlaminar bonding strength [14]
Structural Damage	Extreme loads or cumulative fatigue damage [15]
Special Environmental Damage	Lightning strikes, freezing [16]

**Table 2:** Advantages and disadvantages identified by various researchers in the application of visual and optical inspection methods in WTBs.

Authors	Method	Strengths	Weaknesses
Khadka et al. [32]	DIC and Point Tracking (PT) technology based on UAV platform, combined with dynamic stitching algorithm for vibration monitoring of rotating WTBs.	Combining DIC with UAV for dynamic monitoring of rotating structures; dynamic stitching technology breaks the field-of-view limitation for large-size blade measurement.	Challenges in creating speckle patterns; high complexity in data processing.
Stałowska et al. [33]	Using Phase-Shifting TLS technology combined with photogrammetry and spectral analysis for WTB defect detection.	Performance comparable to photogrammetry in detecting most surface defects, with less influence from weather conditions.	Lower accuracy in detecting blade edge defects, mainly affected by laser spot size and incident angle.
Chen et al. [34]	Solving camera internal parameters through standard calibration plate, eliminating barrel/pincushion distortion of short-focus CCD lenses, improving image geometric accuracy.	No need to implant sensors inside the blade, suitable for real-time monitoring in harsh environments, reducing maintenance difficulty.	Affected by environment and speed; relies on artificial markers.
Yu et al. [35]	Information enhancement and filtering of feature maps through dynamic activation mapping, alleviating information loss in traditional pooling operations, improving detection capability for small target defects.	Strong adaptability to few-shot learning; improved detection accuracy.	Relies on relevant task data; complexity of dynamic mapping; limited industrial practicality.
Guo et al. [36]	Proposed a hierarchical deep learning damage identification framework, combining Haar–AdaBoost region proposal with CNN classification, achieving detection and classification of surface damage on WTBs.	High accuracy and strong robustness; high efficiency and low computational cost.	Information loss from image scaling; reliance on annotation data quality.
Ozbek et al. [37]	Using four CCD cameras and Class Activation Mapping technology to generate soft mask maps, overlaying them on deep feature maps, enhancing task-relevant information and filtering redundant background, alleviating information loss in traditional pooling operations.	Strong adaptability to few-shot learning; enhanced detection capability for small defects; high training efficiency.	Sensitivity to task differences; single defect category; limited ability to distinguish between highly similar defect and non-defect areas.
Poozesh et al. [38]	Using 3D-DIC and 3D-PT technology, combined with multi-camera dynamic spatial data stitching method, achieving full-field displacement, strain, and modal parameter measurement of large WTBs.	Full-field non-contact measurement; efficiency and cost advantages; dynamic stitching extends field of view.	Environmental sensitivity; trade-off between field of view and resolution; limitations in dynamic measurement; large data computation and susceptibility to noise interference.
Wu et al. [39]	Using 3D-DIC technology for WTB health monitoring.	Non-contact full-field measurement; resistance to large deformation and rotation; strong field applicability.	Equipment dependency and data volume limitations; sensitivity to environmental interference; limitations in defect detection.

**Table 3:** Advantages and disadvantages identified by various researchers in the application of acoustic testing methods in WTBs.

Authors	Method	Strengths	Weaknesses
Pang et al. [40]	Method using artificial acoustic source simulation & time difference localization & attenuation characteristic analysis, providing basic data for AE detection of WTB resin matrix.	Simple operation; good localization accuracy; stable and repeatable.	Single simulation scenario; lower localization accuracy in edge areas; insufficient attenuation compensation.
Zhao et al. [41]	Using AE technology combined with wavelet analysis research method to achieve material damage monitoring of WTBs.	High sensitivity; strong anti-interference ability; significant time-domain characteristics.	Significant time-domain characteristics; experimental condition limitations; lack of localization capability.
Tang et al. [43]	Defining “AE event” as pulse signals received by 4 sensors within the propagation time difference range of damage source, calculating signal arrival time difference through triangulation method to determine damage location.	High sensitivity and localization accuracy; strong anti-interference capability; strong practicality.	Limited sensor coverage area; signal attenuation and mode conversion; dependency on multi-sensor synchronization.
Mielke et al. [44]	Using Discrete Wavelet Transform (DWT) for time–frequency feature extraction of acoustic emission signals.	High sensitivity; strong anti-interference; accurate time–frequency feature extraction.	Signal processing complexity; experimental condition limitations.
Zhao et al. [45]	Using semi-analytical finite element method to analyze AE wave propagation characteristics in key structures.	High-precision localization; strong robustness; high practicality.	Dependency on structural prior information; boundary reflection interference; computational complexity.
Yan et al. [46]	Using π-phase-shifted fiber Bragg grating-based acoustic emission detection technology combined with wavelet packet decomposition and empirical mode decomposition fusion denoising method to achieve crack detection in WTBs.	High sensitivity and anti-interference; precise denoising; distinguishable damage features.	Temperature sensitivity; experimental scenario limitations; denoising complexity.
Jiang et al. [47]	Achieving precise identification of WTB damage through AE technology combined with machine learning.	Early damage detection capability; multi-parameter fusion analysis.	Signal interference; small sample limitations; scaled model deviation.
Wang et al. [48]	Obtaining characteristic frequencies of different damage types through AE technology combined with cluster analysis and wavelet transform.	Real-time and dynamic monitoring; high sensitivity; strong applicability to complex structures.	High experimental environment requirements; complex data analysis; difficulty in quantifying damage degree.
Samareh-Mousavi et al. [49]	Using linear localization array method for acoustic emission detection; identifying delamination areas through acoustic event quantity and energy distribution.	Early damage sensitivity; relatively high localization accuracy.	Limited spatial resolution; difficulty in deep damage identification; sensitive to environmental interference.
Benzon et al. [50]	Achieving efficient analysis of AE signals through PCA dimensionality reduction and cluster analysis, verifying the feasibility of full-scale blade damage monitoring.	Full-scale structure applicability; multi-dimensional data fusion; digital twin potential.	Sensor layout dependency; frequency–damage mechanism mapping ambiguity; multi-damage signal interference.
Janeliukstis et al. [51]	Achieving early warning of blade failure before destruction and damage area localization by fusing acoustic emission and strain signals, extracting a series of damage-sensitive features.	High sensitivity; real-time monitoring; multi-feature fusion.	Severe signal attenuation; features affected by setup; complex data processing.
Tsai et al. [55]	Achieving efficient blade surface damage detection through acoustic signal time–frequency analysis and physically driven CNN model, but its binary classification limitations and environmental dependency still need a breakthrough. Future developments can combine multi-modal fusion, intelligent signal enhancement and lightweight design to promote practical application in wind power operation and maintenance.	High accuracy and robustness; small sample adaptability; low cost.	Only supports binary classification; environmental dependency; high-frequency signal dependency.
Solimine et al. [52]	Passive acoustic damage detection method based on internal microphones, using natural environmental excitation and periodic measurement windows through limited distributed microphone network inside the blade, monitoring trend changes, shifts or peaks in sound pressure level in blade cavity, combined with speech processing technology to extract features.	Non-invasive and low complexity; strong adaptability to passive excitation; high sensitivity and multi-damage type detection.	High sensitivity to environmental interference; damage mode limitations; data label dependency.
Beale et al. [53]	Passive acoustic damage detection method based on flow-induced acoustic excitation; core principle is to identify damage by monitoring changes in structural acoustic transmission characteristics of cavity under external airflow excitation.	Non-contact and global monitoring; low cost and easy deployment; high sensitivity.	Insufficient sensitivity in low-frequency bands; limitations in small damage detection; environmental dependency.
Poozesh et al. [54]	Non-contact structural health monitoring method based on acoustic microphone array beamforming technology.	Non-contact and high sensitivity; high spatial resolution; strong anti-interference capability.	Limited by array configuration; large damage masking small damage; strong frequency dependency.
Beale et al. [57]	Achieving damage identification of complex structures through combination of internal acoustic excitation and external acoustic pressure response measurement, using changes in acoustic transmittance of structural cavity boundaries to identify damage.	High sensitivity; non-contact and low sensor requirements; strong anti-environmental interference.	Detection distance limitations; environmental noise interference; sensor position dependency.
Zhang et al. [58]	Using passive acoustic detection method, detecting cracks and debonding defects by analyzing aeroacoustic noise generated by WTB trailing edge faults.	Non-contact monitoring; fast calculation speed; significant high-frequency characteristics.	Sensitive to environmental interference; semi-empirical model simplification errors; grid and computational costs.

**Table 4:** Advantages and disadvantages of vibration-based monitoring in WTB applications as reported by various researchers.

Authors	Method	Strengths	Weaknesses
Fremmelev et al. [61]	Using active vibration data-based features to detect damage initiation and progression in wind turbine blades during fatigue testing.	High sensitivity; strong anti-interference capability; large-range monitoring.	Frequency range dependency; complex component selection; sensitive to sensor position.
Tcherniak et al. [62]	Active vibration excitation combined with semi-supervised learning and mid-frequency signal optimization to detect small 15 cm defects in operating wind turbine blades.	Good balance between high sensitivity and practicality; strong adaptability of semi-supervised learning; strong environmental robustness.	High dependency on sensor configuration; environmental interference still exists; hardware cost and maintenance challenges.
Fitzgerald et al. [63]	Using Short-Time Fourier Transform (STFT) as the core vibration detection method, tracking time–frequency characteristic changes in blade vibration signals to identify damage.	Balanced time–frequency resolution; no additional sensors required; easy integration into existing condition monitoring systems.	Window function limitations; modal simplification errors; sensitive to environmental interference.
Wang et al. [64]	Damage detection method combining finite element method-based dynamic analysis (modal analysis and dynamic response analysis) and Mode Shape Difference Curvature information.	High-precision localization and quantitative assessment; applicable to blades of different sizes and materials; non-destructive and low-cost.	High dependency on model accuracy; difficulty in identifying high-frequency modes of large blades; curvature calculation affected by noise.
Oliveira et al. [65]	Using Multiple Linear Regression model to correct natural frequencies, eliminating effects of wind speed, temperature, rotational speed and other factors, obtaining residual features sensitive to structural damage.	Simultaneous detection of multiple-component damage; reliable long-period data; simple system structure.	Modal identification limitations; insufficient sensitivity to blade damage; insufficient consideration of dynamic effects.
Pacheco-Chérrez et al. [66]	Using OMA-based vibration detection method combined with frequency-domain decomposition algorithm to extract modal parameters and comparing modal characteristics of intact and damaged blades through Mode Shape Difference index to achieve crack damage localization and identification.	No need for known excitation signals; low cost and practicality; high-precision localization capability.	Dependent on high-quality response signals; only applicable to specific damage types; differences between numerical simulation and actual scenarios.
Kim et al. [67]	Using numerical sensors to collect acceleration signals of floating offshore WTBs and blades in dynamic wind fields, using OMA to extract modal parameters, and achieving structural health monitoring by comparing modal differences between intact and damaged states.	No artificial excitation required; curvature mode shapes sensitive to damage; consideration of fully coupled effects.	Dependent on large number of sensors; low-frequency modes affected by floating body motion; idealization of numerical signals.
Rezamand et al. [68]	Decomposing signals through multi-resolution analysis to extract time–frequency-domain features of fault vibration signals.	Effective capture of transient fault characteristics; sensitive to early minor faults; capable of real-time tracking of system dynamic changes.	Dependent on wavelet basis function selection; computational complexity; susceptible to noise interference.
Ogaili et al. [69]	Time-domain feature extraction & feature selection & machine learning classification method based on vibration signals.	Coverage of multiple fault types; efficient feature optimization; simple calculation of time-domain features.	Limited feature dimensions; dataset singularity; insufficient algorithm generalization.
Fremmelev et al. [61]	Using active vibration data-based features to detect damage initiation and progression in wind turbine blades during fatigue testing.	High sensitivity; strong anti-interference capability; large-range monitoring.	Frequency range dependency; complex component selection; sensitive to sensor position.
Tcherniak et al. [62]	Active vibration excitation combined with semi-supervised learning and mid-frequency signal optimization to detect small 15 cm defects in operating wind turbine blades.	Good balance between high sensitivity and practicality; strong adaptability of semi-supervised learning; strong environmental robustness.	High dependency on sensor configuration; environmental interference still exists; hardware cost and maintenance challenges.
Fitzgerald et al. [63]	Using Short-Time Fourier Transform (STFT) as the core vibration detection method, tracking time–frequency characteristic changes of blade vibration signals to identify damage.	Balanced time–frequency resolution; no additional sensors required; easy integration into existing condition monitoring systems.	Window function limitations; modal simplification errors; sensitive to environmental interference.
Wang et al. [64]	Damage detection method combining finite element method-based dynamic analysis (modal analysis and dynamic response analysis) and Mode Shape Difference Curvature information.	High-precision localization and quantitative assessment; applicable to blades of different sizes and materials; non-destructive and low-cost.	High dependency on model accuracy; difficulty in identifying high-frequency modes of large blades; curvature calculation affected by noise.
Oliveira et al. [65]	Using Multiple Linear Regression model to correct natural frequencies, eliminating effects of wind speed, temperature, rotational speed and other factors, obtaining residual features sensitive to structural damage.	Simultaneous detection of multiple-component damage; reliable long-period data; simple system structure.	Modal identification limitations; insufficient sensitivity to blade damage; insufficient consideration of dynamic effects.
Pacheco-Chérrez et al. [66]	Using OMA-based vibration detection method, combined with frequency-domain decomposition algorithm to extract modal parameters, and comparing modal characteristics of intact and damaged blades through Mode Shape Difference index to achieve crack damage localization and identification.	No need for known excitation signals; low cost and practicality; high-precision localization capability.	Dependent on high-quality response signals; only applicable to specific damage types; differences between numerical simulation and actual scenarios.
Kim et al. [67]	Using numerical sensors to collect acceleration signals of floating offshore WTBs and blades in dynamic wind fields, using OMA to extract modal parameters, and achieving structural health monitoring by comparing modal differences between intact and damaged states.	No artificial excitation required; curvature mode shapes sensitive to damage; consideration of fully coupled effects.	Dependent on large number of sensors; low-frequency modes affected by floating body motion; idealization of numerical signals.
Rezamand et al. [68]	Decomposing signals through multi-resolution analysis to extract time–frequency-domain features of fault vibration signals.	Effective capture of transient fault characteristics; sensitive to early minor faults; capable of real-time tracking of system dynamic changes.	Dependent on wavelet basis function selection; computational complexity; susceptible to noise interference.
Ogaili et al. [69]	Time-domain feature extraction & feature selection & machine learning classification method based on vibration signals.	Coverage of multiple fault types; efficient feature optimization; simple calculation of time-domain features.	Limited feature dimensions; dataset singularity; insufficient algorithm generalization.

**Table 5:** Advantages and disadvantages identified by various researchers in the application of ultrasonic testing methods in WTBs.

Authors	Method	Strengths	Weaknesses
Yang et al. [71]	Using characteristics of nonlinear responses caused by damage detected by high-frequency ultrasound to inspect wind turbine blade damage.	Accurate localization; low attenuation characteristics; strong anti-interference capability.	Insensitive to large structures; insufficient excitation energy; background nonlinear interference.
Muñoz et al. [22]	Ultrasonic testing technology based on Macro-Fiber Composite sensors, combined with wavelet transform signal processing and multilayer perceptron neural network pattern recognition, to achieve detection and classification of mud accumulation thickness on wind turbine blade surfaces.	High sensor performance compatibility; high accuracy.	Sample limitations; frequency dependency.
Muñoz et al. [72]	Using ultrasonic guided waves combined with Macro-Fiber Composite sensors, collecting signals through pitch–catch mode.	Long-distance detection capability; high sensitivity; anti-noise interference.	Signal complexity; temperature sensitivity; sensor placement limitations.
Burnham et al. [73]	Using ultrasonic guided waves and acoustic emission technology for integrity monitoring of composite structures in wind turbine blades.	Large-area monitoring capability; early defect detection; non-invasive, remote monitoring possible.	High attenuation characteristics; anisotropic interference; multi-mode and dispersion.
Kang et al. [74]	Combined method using laser ultrasonic excitation & piezoelectric flexible line sensor detection, providing new ideas for non-destructive testing of curved composite structures such as wind turbine blades.	Non-contact, large-area detection; adaptable to curved surface detection; high sensitivity and resolution.	Signal noise interference; sensor placement limitations; insufficient environmental adaptability.
Sun et al. [75]	Deploying contact ultrasonic probes on negative-pressure adsorption vehicles through UAVs to achieve in-service internal defect detection of wind turbine blades.	Online detection; high detection accuracy.	Environmental interference; balance of detection efficiency.
Oliveira et al. [76]	Using ultrasonic non-destructive testing method combined with signal processing and machine learning technology to identify defects in wind turbine blades.	No defect samples required for training; high accuracy.	Poor stability in dry spot defect detection; reliance on signal preprocessing quality.
Raisutis et al. [77]	Using a new method based on ultrasonic guided-wave phase-velocity changes to achieve detection, localization and size estimation of artificial defects in wind turbine blades.	Sensitive to defects; resistant to attenuation interference; no calibration required.	Computational complexity; sensitive to sensor spacing; limited to specific defect types.
Li et al. [78]	Achieving precise localization of hidden defects inside wind turbine blades by decomposing superimposed ultrasonic echoes caused by thin blade webs and extracting time-delay parameters.	Strong anti-interference capability; high-precision localization.	High computational complexity; strong dependency on basic waveform library.
Caminero et al. [79]	Using phased array ultrasonic testing technology, employing C-scan imaging and S-scan imaging for composite material detection.	High-precision localization; visualization of damage propagation; wide applicability.	Insufficient accuracy in identifying defect shape and size; limited detection capability for weak reflection defects.
Zhang et al. [80]	Using defect test block-based ultrasonic phased array testing technology combined with C-scan imaging technology to achieve defect detection.	High detection efficiency; strong internal defect identification capability; good flexibility.	Material penetration limitations; high equipment dependency.
Sun et al. [81]	Based on Fermat spiral array ultrasonic phased array, combined with total focusing imaging technology to achieve crack detection.	High detection sensitivity; low interference noise.	High manufacturing difficulty; high computational complexity.

**Table 6:** Advantages and disadvantages identified by various researchers in the application of thermal imaging technology in WTBs.

Authors	Method	Strengths	Weaknesses
Sanati et al. [82]	Effectively achieving subsurface defect detection in WTBs through active and passive thermography combined with image processing.	Active thermography: high controllability, high sensitivity; passive thermography: low cost, practical.	Active thermography: requires external heat source; passive thermography: low image quality.
He et al. [83]	Applying instantaneous thermal pulses to the surface of glass fiber-reinforced plastic WTB specimens using high-energy flash lamps, recording surface temperature decay process with infrared cameras.	High detection efficiency; accurate defect localization.	Thickness limitations; surface treatment required; thermal diffusion interference.
Tao et al. [84]	Applying short-pulse heating to blade surfaces using flash lamps, monitoring surface temperature field changes with infrared cameras, thereby detecting WTB defects.	Large-area detection; multiple defect types detectable.	Limited deep defect detection; sensitive to environmental interference.
Doroshtnasir et al. [85]	Using differential thermal images to eliminate environmental interference in WTB thermography, achieving defect detection.	Long-distance detection; strong interference suppression capability.	Dependent on blade consistency; image registration errors.
Jensen et al. [86]	Using long-pulse thermography combined with Principal Component Analysis as the core detection method for subsurface defect detection in WTB leading edges.	High defect detection capability; adaptability to curved surfaces and coatings.	Excitation time dependent on defect depth; low data acquisition efficiency.
Manohar et al. [87]	Using statistically enhanced lock-in thermography technology, combined with multivariate outlier analysis and image processing algorithms, to detect delamination defects in WTBs.	Overcoming blind frequency effect; high defect contrast.	Limited detection depth; high data processing complexity.
Hwang et al. [88]	Generating thermal waves on WTB surfaces using continuous-wave line lasers, simultaneously recording thermal wave propagation with infrared cameras, achieving WTB defect detection.	Full-area detection; high precision and anti-interference capability.	Strict laser intensity control; dependent on stable rotation speed; limited detection depth.
Hwang et al. [89]	Using continuous line laser scanning thermography system, mechanically scanning line laser beams and synchronously collecting thermal wave responses, achieving remote detection of internal delamination in WTBs.	Remote non-contact detection; fast speed; high sensitivity.	Limited spatial resolution; laser power and material compatibility required.
Zhang et al. [90]	Using prolonged continuous heating to allow heat penetration into deep material layers, capturing surface temperature field changes with infrared cameras, combined with 3D heat conduction model analysis of defect depth.	Deep defect detection; high accuracy.	Model assumption limitations; low efficiency.
Yang et al. [91]	Introducing photothermal thermal wave radar non-destructive imaging technology into induction infrared thermography, achieving delamination defect detection in WTBs.	High signal-to-noise ratio; full-field, non-contact detection.	Limited detection depth; non-uniform near-field heating; insufficient 3D imaging capability.
Cheng et al. [92]	Applying high-frequency electromagnetic pulses to conductive materials, inducing eddy currents to generate Joule heat inside the material, then using infrared thermal imagers to capture temperature changes in defect areas, achieving defect detection.	Non-contact, large-area rapid detection; deep penetration capability.	Limited sensitivity; coil position dependency; high experimental condition requirements.
Liang et al. [93]	Using eddy current pulsed thermography combined with wavelet transform to detect low-energy impact damage.	Non-contact and large-area detection; multi-physics field coupling advantages; capable of detecting weak defects.	High data processing complexity; time window dependency.
Reta et al. [94]	Using long-pulse active thermography combined with computer vision and machine learning technology to achieve subsurface defect detection in composite materials.	Non-destructive; high defect localization accuracy; multi-defect type recognition capability.	Limited detection depth; low image resolution.
Chon et al. [95]	Thermal imaging based on synthetic data and conversion modules to achieve wind turbine blade defect detection.	Few samples required; strong adaptability.	Poor detection effect for shallow defects; dependent on thermal equilibrium stage data.

**Table 7:** Performance comparison of major NDT technologies for WTBs.

Technology Category	Main Inspection Objects	Detection Precision	Cost	Applicable	Main Advantages	Main Limitations
Visual/Optical	Surface cracks, corrosion, deformation	√√	√	Onshore: √√√ Offshore: √	Intuitive, non-contact, full-field measurement, efficient (combined with UAV)	Cannot detect internal defects, affected by lighting/weather
Acoustic Emission	Dynamic cracks, delamination, fiber breakage	√√√	√√	Onshore: √√√ Offshore: √√	Real-time monitoring, global monitoring, high sensitivity	Requires many sensors, complex localization, susceptible to noise interference
Vibration Analysis	Overall stiffness loss, large-scale damage	√	√√	Onshore: √√ Offshore: √	Enables long-term online monitoring, mature technology	Sensitive to environment & operational conditions, difficult to detect small defects
Ultrasonic	Internal pores, delamination, debonding	√√√	√√√	Onshore: √√ Offshore: √	Sensitive to internal defects, strong quantification capability	Requires couplant, slow inspection speed, challenging for complex geometries
Thermal Imaging	Near-surface delamination, debonding, water ingress	√√	√√	Onshore: √√√ Offshore: √	Non-contact, fast imaging, large-area coverage	Affected by surface emissivity & ambient temperature, limited penetration depth

**Note:** The symbols used in this table are defined as follows: Detection precision: √√√ = High precision; √√ = Medium precision; √ = Low precision. Cost: √√√ = High; √√ = Medium; √ = Low. Applicability: √√√ = Highly suitable; √√ = Suitable; √ = Limited suitability.

**Table 8:** Comparison of emerging intelligent inspection technologies and data-driven methods for WTBs.

Technology/Method Category	Key Techniques/Algorithms	Main Applications	Strengths	Limitations
Machine Learning (Supervised)	SVM, KNN, decision trees, ANN	Fault classification, damage type recognition	High accuracy with labeled data; mature algorithms	Requires large labeled datasets; limited generalization across turbines
Deep Learning (Vision-Based)	CNN, YOLO, MobileNet, MI-YOLO	Surface crack detection, defect recognition from UAV images	Powerful feature extraction; high detection accuracy; automated	Requires large training data; black-box nature; computational cost
Unsupervised/Semi-Supervised Learning	PCA, autoencoder, K-means, self-supervised learning	Anomaly detection, health representation learning	No/limited labels required; detects unknown patterns	Difficult to interpret results; risk of false alarms
Advanced Signal Processing	Wavelet transform, WPD, time–frequency analysis	Feature extraction from AE/vibration/acoustic signals	Enhances SNR; captures transient/non-stationary features	Feature selection complexity; sensitive to noise
SCADA Data Mining & Digital Twin	Correlation analysis, autoencoder, pattern recognition	Fleet-level monitoring, remaining life prediction	Low-cost; leverages existing data; enables predictive maintenance	Data quality issues; requires domain expertise for modeling
Robotic Platforms	UAV with cameras/thermal imagers, climbing robots with multi-sensors	Automated external/internal inspection	Safe access to difficult areas; multi-sensor integration; high efficiency	Safe access to difficult areas; multi-sensor integration; high efficiency

## Data Availability

All data reported in this review are available from the referenced original sources.

## Supplementary Materials

The following supporting information can be downloaded at: https://www.mdpi.com/article/10.3390/s26061773/s1, Table S1: PRISMA 2020 Checklist used in the current systematic review study. Ref. [133] cited in Supplementary Materials.

## Abbreviations

The following abbreviations are used in this manuscript:

AE	Acoustic Emission
CCD	Charge-Coupled Device
CFRP	Carbon Fiber-Reinforced Polymer
CNN	Convolutional Neural Network
DIC	Digital Image Correlation
GFRP	Glass Fiber-Reinforced Polymer
MI-YOLO	Multivariate Information YOLO
NDT	Non-Destructive Testing
NREL	National Renewable Energy Laboratory
O&M	Operation and Maintenance
OMA	Operational Modal Analysis
PCA	Principal Component Analysis
PDF	Probability Density Function
PT	Point Tracking
RE	Reconstruction Error
RPCA	Recursive Principal Component Analysis
SCADA	Supervisory Control and Data Acquisition
SHM	Structural Health Monitoring
TLS	Terrestrial Laser Scanning
TWR	Thermal Wave Radar
UAV	Unmanned Aerial Vehicle
WPD	Wavelet Packet Decomposition
WTB	Wind Turbine Blade
YOLO	You Only Look Once
