# Supplementary material for: Advances, Challenges, and Recommendations for Non-Destructive Testing Technologies for Wind Turbine Blade Damage: A Review of the Literature from the Past Decade
Source: Sensors (Basel). 2026 Mar 11;26(6):1773. doi: 10.3390/s26061773 (PMC13030368; doi:10.3390/s26061773)
Supplement: Supplementary file 1 [file sensors-26-01773-s001.zip › sensors-4159627-supplementary.pdf]

1. PRISMA 2020 Main Checklist

Topic	No.	Item	Location where item is reported
**TITLE**			
**Title**	1	Identify the report as a systematic review.	Line 2-4
**ABSTRACT**			
**Abstract**	2	See the PRISMA 2020 for Abstracts checklist	
**INTRODUCTION**			
**Rationale**	3	Describe the rationale for the review in the context of existing knowledge.	1.1 Research Background and Significance
**Objectives**	4	Provide an explicit statement of the objective(s) or question(s) the review addresses.	1.4
**METHODS**			

Topic	No.	Item	Location where item is reported
**Eligibility criteria**	5	Specify the inclusion and exclusion criteria for the review and how studies were grouped for the syntheses.	Inclusion Criteria: Studies were considered for inclusion if they met all of the following criteria: Topic Relevance: Focused on non-destructive testing (NDT) or structural health monitoring (SHM) methods for the detection, diagnosis, or monitoring of damage in wind turbine blades. Content Type: Presented original research, a comprehensive review, or a significant case study with clear methodological insights. This included peer-reviewed journal articles, conference proceedings, and authoritative technical reports. Temporal Scope: Published within the past decade (approximately 2014–2024), to ensure the review captured recent technological advances. Seminal works outside this window were cited selectively for foundational context. Exclusion Criteria: Studies were excluded if they: Focused primarily on NDT/SHM for other wind turbine components (e.g., gearbox, tower, foundation) without direct application or transferable principles to blades. Were solely about blade design, manufacturing, materials (without a primary focus on inspection/damage detection), or aerodynamic performance. Were published in non-peer-reviewed sources (e.g., most commercial websites, unverified blogs) or were patents, unless they introduced a highly novel methodological concept. Grouping for Synthesis: Included studies were grouped for narrative synthesis based on the primary NDT technology family they addressed (e.g., visual/optical, acoustic, vibration, ultrasonic, thermographic) and their methodological approach (traditional vs. intelligent/data-driven). This thematic grouping directly structured the review (Sections 2.1–2.5 and Section 3).
**Information sources**	6	Specify all databases, registers, websites, organisations, reference lists and other sources searched or consulted to identify studies. Specify the date when each source was last searched or consulted.	Web of Science Core Collection (searched January 2026), Scopus (searched January 2026), Google Scholar (searched January 2026), and reference lists of key publications

Topic	No.	Item	Location where item is reported
**Search strategy**	7	Present the full search strategies for all databases, registers and websites, including any filters and limits used.	We conducted a comprehensive literature search using keyword-based queries in multiple academic databases, including Web of Science.The search query was:<br>TS = (("wind turbine blade*") AND (<br>("non-destructive" OR "NDT") OR<br>("structural health monitoring" OR "SHM") OR<br>("damage detection" OR "damage identification") OR<br>("acoustic emission" OR "AE") OR<br>("ultrasonic" OR "ultrasound" OR "guided wave" OR "phased array") OR<br>("vibration" OR "modal analysis" OR "OMA" OR "operational modal analysis") OR<br>("thermography" OR "infrared" OR "thermal imaging" OR "IRT") OR<br>("visual inspection" OR "optical" OR "photogrammetry" OR "DIC" OR "digital image correlation") OR<br>("machine learning" OR "deep learning" OR "CNN" OR "convolutional neural network" OR "neural network" OR "YOLO" OR "artificial intelligence" OR "AI") OR<br>("robot" OR "robotics" OR "UAV" OR "drone" OR "unmanned aerial vehicle" OR "climbing robot" OR "crawler")<br>))Time range: The search covered publications from January 2010 to December 2025, capturing the most recent decade of research.<br>Document type: We limited the search to "Article" document types to ensure peer-reviewed original research.<br>Search results: The initial search yielded 1962 publications, which formed the basis for our bibliometric analysis and literature synthesis.

Topic	No.	Item	Location where item is reported
**Selection process**	8	Specify the methods used to decide whether a study met the inclusion criteria of the review, including how many reviewers screened each record and each report retrieved, whether they worked independently, and if applicable, details of automation tools used in the process.	The literature for this review was initially identified through keyword searches in major databases (e.g., Web of Science, Scopus). The selection was then performed by the authors, who screened titles, abstracts, and subsequently full texts to identify studies that focused on NDT/SHM methods for wind turbine blade damage, published within the last ten years. Studies were included based on their relevance to the review's objectives of comparing technologies, discussing challenges, and outlining future trends.
**Data collection process**	9	Specify the methods used to collect data from reports, including how many reviewers collected data from each report, whether they worked independently, any processes for obtaining or confirming data from study investigators, and if applicable, details of automation tools used in the process.	The data collection process focused on extracting qualitative and descriptive information relevant to the review's themes. Key information was collected on the following aspects from each relevant publication: the NDT/SHM technique used, its application context (laboratory/field), the type of damage detected, reported performance metrics (e.g., accuracy, sensitivity), and the study's main conclusions regarding limitations and future work. This information was thematically organized to facilitate comparative analysis and synthesis across different technologies.
**Data items**	10a	List and define all outcomes for which data were sought. Specify whether all results that were compatible with each outcome domain in each study were sought (e.g. for all measures, time points, analyses), and if not, the methods used to decide which results to collect.	Data were collected on the reported performance outcomes of each NDT technology, primarily focusing on their damage detection capability, accuracy, and practical applicability in wind turbine blade monitoring contexts.
	10b	List and define all other variables for which data were sought (e.g. participant and intervention characteristics, funding sources). Describe any assumptions made about any missing or unclear information.	To enable a critical analysis of the literature's context and evidence strength, we extracted supplementary variables including: (1) the specific configuration of the technology described; (2) the study's experimental or application setting (lab/field/simulation); and (3) the scale and nature of the validation performed. This allowed us to assess the translational readiness of the technologies discussed.

Topic	No.	Item	Location where item is reported
**Study risk of bias assessment**	11	Specify the methods used to assess risk of bias in the included studies, including details of the tool(s) used, how many reviewers assessed each study and whether they worked independently, and if applicable, details of automation tools used in the process.	Not Applicable
**Effect measures**	12	Specify for each outcome the effect measure(s) (e.g. risk ratio, mean difference) used in the synthesis or presentation of results.	Not Applicable
**Synthesis methods**	13a	Describe the processes used to decide which studies were eligible for each synthesis (e.g. tabulating the study intervention characteristics and comparing against the planned groups for each synthesis (item 5)).	For the narrative synthesis, studies were grouped into coherent thematic sections based on the core non-destructive testing technology they primarily addressed. This grouping directly corresponds to the organizational structure of the review (Sections 2.1 through 2.5, and Section 3), enabling a focused analysis and comparison within each technological domain.
	13b	Describe any methods required to prepare the data for presentation or synthesis, such as handling of missing summary statistics, or data conversions.	Not Applicable
	13c	Describe any methods used to tabulate or visually display results of individual studies and syntheses.	Table 2, 3, 4, 5, 6, 7
	13d	Describe any methods used to synthesize results and provide a rationale for the choice(s). If meta-analysis was performed, describe the model(s), method(s) to identify the presence and extent of statistical heterogeneity, and software package(s) used.	Not Applicable
	13e	Describe any methods used to explore possible causes of heterogeneity among study results (e.g. subgroup analysis, meta-regression).	Not Applicable

Topic	No.	Item	Location where item is reported
	13f	Describe any sensitivity analyses conducted to assess robustness of the synthesized results.	Not Applicable
**Reporting bias assessment**	14	Describe any methods used to assess risk of bias due to missing results in a synthesis (arising from reporting biases).	Not Applicable
**Certainty assessment**	15	Describe any methods used to assess certainty (or confidence) in the body of evidence for an outcome.	Not Applicable
**RESULTS**			
**Study selection**	16a	Describe the results of the search and selection process, from the number of records identified in the search to the number of studies included in the review, ideally using a flow diagram.	A systematic literature search was conducted in Web of Science Core Collection using a comprehensive search strategy (see Item 7). This was supplemented by additional searches in Scopus, Google Scholar, and manual screening of reference lists. This iterative and expertise-driven approach yielded a final corpus of 132 references, all of which were deemed pertinent to the review's objectives of examining advances, challenges, and future trends in NDT for wind turbine blades.
	16b	Cite studies that might appear to meet the inclusion criteria, but which were excluded, and explain why they were excluded.	A list of studies excluded after full-text review is not provided, as the selection for this narrative review was based on thematic relevance and contribution to the synthesis rather than a binary eligibility screening process.
**Study characteristics**	17	Cite each included study and present its characteristics.	A total of 132 references are cited throughout the text, but they are not listed collectively.
**Risk of bias in studies**	18	Present assessments of risk of bias for each included study.	Not Applicable
**Results of individual studies**	19	For all outcomes, present, for each study: (a) summary statistics for each group (where appropriate) and (b) an effect estimate and its precision (e.g. confidence/credible interval), ideally using structured tables or plots.	Not applicable

Topic	No.	Item	Location where item is reported
**Results of syntheses**	20a	For each synthesis, briefly summarise the characteristics and risk of bias among contributing studies.	For each technological synthesis presented in Sections 2.1–2.5 and Section 3, the contributing studies share common characteristics: they are predominantly experimental or methodological studies published in peer-reviewed journals and conference proceedings over the past decade. The evidence base includes a mix of proof-of-concept laboratory experiments on material coupons or sub-components, numerical simulations, and a smaller proportion of full-scale blade or field validation studies. As noted in Item 11, a formal assessment of the risk of bias was not conducted for this narrative review. However, the narrative synthesis inherently considers and discusses the implications of these methodological variations—such as the limited generalizability of coupon-level results to entire blades or the challenges of environmental control in field studies—when interpreting the findings and evaluating technological readiness.
	20b	Present results of all statistical syntheses conducted. If meta-analysis was done, present for each the summary estimate and its precision (e.g. confidence/credible interval) and measures of statistical heterogeneity. If comparing groups, describe the direction of the effect.	Not applicable
	20c	Present results of all investigations of possible causes of heterogeneity among study results.	Not applicable
	20d	Present results of all sensitivity analyses conducted to assess the robustness of the synthesized results.	Not applicable
**Reporting biases**	21	Present assessments of risk of bias due to missing results (arising from reporting biases) for each synthesis assessed.	Not applicable
**Certainty of evidence**	22	Present assessments of certainty (or confidence) in the body of evidence for each outcome assessed.	Not applicable

Topic	No.	Item	Location where item is reported
**DISCUSSION**			
**Discussion**	23a	Provide a general interpretation of the results in the context of other evidence.	Section Conclusion
	23b	Discuss any limitations of the evidence included in the review.	Section 4.1

23c	Discuss any limitations of the review processes used.	Several limitations inherent to the methodology of this narrative review should be acknowledged: 1. Non-Systematic Search: The literature identification process, while comprehensive within its scope, was not a systematic search across all possible databases with a predefineSeveral limitations inherent to the methodology of this review should be acknowledged:<br><br>1. Database Coverage and Search Limitations: Although a comprehensive and structured search was conducted in the Web of Science Core Collection using an enhanced keyword strategy covering traditional and emerging NDT techniques, the search was limited to a single primary database. While supplementary searches in other databases (e.g., Scopus, Google Scholar) and manual screening of reference lists were performed to mitigate this limitation, it is possible that some relevant studies, particularly those published in non-indexed journals or in languages other than English, may have been inadvertently omitted.<br><br>2. Study Selection and Thematic Grouping: The selection of studies and their thematic grouping into technology categories (e.g., acoustic emission, ultrasonic testing, machine learning) were based on the authors' expertise and judgment. Although this approach enables a nuanced and critical synthesis tailored to the review's objectives, it introduces an element of subjectivity that differs from the independent, dual-reviewer processes typically employed in systematic reviews. To enhance transparency, the selection process has been documented in detail in the PRISMA flow diagram.<br><br>3. Lack of Formal Quality Appraisal: As this is a narrative review focused on synthesizing technological advances and identifying research trends rather than evaluating intervention effectiveness, we did not employ standardized tools to critically appraise the risk of bias or methodological quality of each included study. Consequently, the synthesis presents and discusses findings from studies of varying rigor without a formal weighting mechanism. However, the narrative discussion inherently considers the

implications of these methodological variations (e.g., laboratory vs. field validation, coupon-level vs. full-scale testing) when interpreting findings and assessing technological readiness.

4. Temporal Scope: By concentrating on literature from 2010 to 2025 to capture recent advances, some foundational or seminal works published before this period may receive less detailed discussion. While key foundational studies were cited where necessary for context, this temporal focus may limit the historical perspective for certain well-established technologies.

These limitations are common to narrative reviews of this scope and nature. We have sought to mitigate their impact by: (1) employing a structured and reproducible search strategy; (2) supplementing database searches with multiple complementary methods; (3) being transparent about our selection and grouping processes; and (4) engaging in critical (rather than merely descriptive) analysis of the included literature. The final corpus of 132 studies (83 from Web of Science and 49 from supplementary sources) provides a robust foundation for the synthesis and conclusions pred and reproducible search string. This may introduce the possibility of selection bias, where some relevant studies, particularly in lesser-known sources or in languages other than English, might have been overlooked. 2. Subjective Study Selection and Synthesis: The selection and thematic grouping of studies were based on the authors' expertise and judgment. Although this allows for a nuanced and critical synthesis, it introduces an element of subjectivity that differs from the independent, dual-reviewer processes used in systematic reviews. 3. Lack of Formal Quality Appraisal: As a narrative review, we did not employ standardized tools to critically appraise the risk of bias or methodological quality of each included study. Therefore, the synthesis presents and discusses findings from studies of varying rigor without a formal weighting mechanism. 4. Focus on Recent Advances: By concentrating on literature from the past decade to highlight advances, some

Topic	No.	Item	Location where item is reported
			foundational or earlier seminal works may receive less detailed discussion, potentially omitting important historical context for certain technologies. These limitations are common to narrative reviews of this kind. We have sought to mitigate their impact by being transparent about our process, aiming for broad coverage within the core theme, and engaging in critical (rather than merely descriptive) analysis of the included literature.
	23d	Discuss implications of the results for practice, policy, and future research.	Section 4.2
**OTHER INFORMATION**			
**Registration and protocol**	24a	Provide registration information for the review, including register name and registration number, or state that the review was not registered.	Not applicable
	24b	Indicate where the review protocol can be accessed, or state that a protocol was not prepared.	Not applicable
	24c	Describe and explain any amendments to information provided at registration or in the protocol.	Not applicable
**Support**	25	Describe sources of financial or non-financial support for the review, and the role of the funders or sponsors in the review.	Section Funding
**Competing interests**	26	Declare any competing interests of review authors.	Section Conflicts of Interest
**Availability of data, code and other materials**	27	Report which of the following are publicly available and where they can be found: template data collection forms; data extracted from included studies; data used for all analyses; analytic code; any other materials used in the review.	Section Data Availability Statement

PRIMSA Abstract Checklist

Topic	No.	Item	Reported?
**TITLE**			
**Title**	1	Identify the report as a systematic review.	Yes
**BACKGROUND**			
**Objectives**	2	Provide an explicit statement of the main objective(s) or question(s) the review addresses.	Yes
**METHODS**			
**Eligibility criteria**	3	Specify the inclusion and exclusion criteria for the review.	No
**Information sources**	4	Specify the information sources (e.g. databases, registers) used to identify studies and the date when each was last searched.	No
**Risk of bias**	5	Specify the methods used to assess risk of bias in the included studies.	Not applicable
**Synthesis of results**	6	Specify the methods used to present and synthesize results.	No
**RESULTS**			
**Included studies**	7	Give the total number of included studies and participants and summarise relevant characteristics of studies.	Yes
**Synthesis of results**	8	Present results for main outcomes, preferably indicating the number of included studies and participants for each. If meta-analysis was done, report the summary estimate and confidence/credible interval. If comparing groups, indicate the direction of the effect (i.e. which group is favoured).	No
**DISCUSSION**			
**Limitations of evidence**	9	Provide a brief summary of the limitations of the evidence included in the review (e.g. study risk of bias, inconsistency and imprecision).	Yes
**Interpretation**	10	Provide a general interpretation of the results and important implications.	Yes
**OTHER**			
**Funding**	11	Specify the primary source of funding for the review.	Yes
**Registration**	12	Provide the register name and registration number.	Not registered

From: Page MJ, McKenzie JE, Bossuyt PM, Boutron I, Hoffmann TC, Mulrow CD, et al. The PRISMA 2020 statement: an updated guideline for reporting systematic reviews. MetaArXiv. 2020, September 14. DOI: 10.31222/osf.io/v7gm2. For more information, visit: www.prisma-statement.org
